# Technical and biological factors driving inter-individual body burden of arsenic species in murine models of human arsenic exposure

**DOI:** 10.1093/toxsci/kfag055

**Published:** 2026-05-19

**Authors:** Lu Wang, Qian Wang, Trenton M Wolfe, Nicholas V Pinkham, Reece Erickson, Masafumi Yoshinaga, Timothy R McDermott, Seth T Walk

**Affiliations:** Department of Microbiology and Cell Biology, Montana State University, Bozeman, MT 59717, United States; Department of Microbiology and Cell Biology, Montana State University, Bozeman, MT 59717, United States; Department of Microbiology and Cell Biology, Montana State University, Bozeman, MT 59717, United States; Department of Microbiology and Cell Biology, Montana State University, Bozeman, MT 59717, United States; Department of Microbiology and Cell Biology, Montana State University, Bozeman, MT 59717, United States; Department of Molecular and Cellular Biology, Kennesaw State University, Kennesaw, GA 30144, United States; Department of Land Resources and Environmental Sciences, Montana State University, Bozeman, MT 59717, United States; Department of Microbiology and Cell Biology, Montana State University, Bozeman, MT 59717, United States

**Keywords:** arsenic, speciation, HPLC-ICPMS, mouse models, risk assessment, diet, microbiome, inter-individual variability

## Abstract

Arsenic is one of the most important environmental toxicants, requiring advanced analytical techniques to resolve individual species. There is little consensus on arsenic speciation methodology for in vivo studies. The objectives of this study were to generate a robust framework for arsenic speciation in murine models of human exposure and evaluate factors influencing the levels of arsenobetaine, inorganic arsenite, dimethylarsinate, monomethylarsonate, and inorganic arsenate resolved by high-performance liquid chromatography (HPLC)–inductively coupled plasma mass spectrometry (ICPMS). Enzyme-assisted digestion by papain vs. pepsin and maceration by bead beating vs. mechanical homogenization were evaluated using chemical standard spiking experiments. Dose-controlled mouse exposures to inorganic arsenite were conducted, and species detected in urine and bladder tissue were compared. Species in stool, liver, and bladder were compared between groups of mice eating a standard vs. purified diet, fasted vs. unfasted mice, and conventional vs. germ-free mice. Finally, between-lab differences in HPLC-ICPMS instrumentation/quantification procedures were evaluated. These comparisons led to several important conclusions, including: Significant conversion of inorganic arsenate to arsenite by papain, significant inorganic arsenate background in bead-beating lysing matrix, significant arsenobetaine in mice eating standard but not purified chow, significant correlation between species detected in urine and bladder, significant correlation of results between laboratories that differed in absolute quantification, and large inter-individual variability between mice of the same treatment group. Finally, diet type and the presence of a microbiome had the largest effect on arsenic species levels. Our results provide a benchmark for evaluating arsenic species in murine models, including adequate sample sizes for powering studies to avoid erroneous conclusions.

## Introduction

Arsenic is a naturally occurring toxic metalloid that is widely distributed in the environment (Wang et al. 2023). The World Health Organization (WHO) estimates over 200 million people worldwide are chronically exposed to arsenic in drinking water above the current maximum contaminant level of 10 µg/l (World Health Organization 2022). Inorganic forms of arsenic (arsenite, iAs^III^ and arsenate, iAs^V^) are the most common species in drinking water and are well-established human carcinogens associated with skin, bladder, lung, and liver cancer (Faita et al. 2013; Hong et al. 2014). Chronic iAs exposure also increases the risk of cardiovascular disorders and diabetes (Faita et al. 2013; Hong et al. 2014). Following ingestion, the toxicity of arsenic is highly dependent on its chemical form, varying as a function of iAs species (pentavalent vs. trivalent) and the degree of methylation and/or thiolation (Moe et al. 2016). Both mammalian and microbial cells can enzymatically convert inorganic arsenic into other species and are therefore important factors affecting toxicity.

Rodent models, particularly mice, are used extensively in toxicology, but specific and broadly accepted methods for processing and evaluating arsenic species in mouse tissues are lacking. For example, studies have exposed lab mice to both forms of iAs in drinking water at levels ranging from 1 µg/l (parts per billion, ppb) to >100 mg/l (parts per million) (Lu et al. 2014b; Barai et al. 2017; Coryell et al. 2018; Chi et al. 2019). Questions that often go unaddressed in studies with murine models are: How were the samples collected/processed, and what arsenic species were generated in vivo? Additionally, it is possible that the diet (chow) and water source (e.g. municipal water) used for lab mice can be a significant and variable source of background arsenic in experiments. Finally, exposed humans and mice both exhibit inter-individual variability in arsenic burden as well as in toxicity outcomes (Coryell et al. 2018), so determining the appropriate sample size needed to conduct rigorous statistical comparisons is important. Experimental evaluation of these factors is needed to help generate more accurate disease risk predictions and understand the impacts of attributable factors.

There are 2 analytical approaches for studying arsenic species in murine models: (i) hydride generation (HG), where arsenicals are converted to volatile arsines and separated by boiling points, and (ii) high-performance liquid chromatographic (HPLC) separation of arsenic species based on the charge or size of analytes. The HG technique has its advantages; however, its primary disadvantage is that it requires customized adaptation to detectors (Styblo et al. 2021), whereas HPLC separation is easily and directly coupled to inductively coupled plasma mass spectrometry (ICPMS) and is highly sensitive for detecting and identifying arsenic species. HPLC-ICPMS has proven to be more broadly used among labs and is the most cited in arsenic exposure research. With respect to animal tissues, a robust protocol for HPLC-ICPMS speciation of arsenic was developed to evaluate the safety of retail chicken exposed to the organoarsenical, Roxarsone (Peng et al. 2014). In addition to quantifying Roxarsone and organoarsenical metabolites of Roxarsone, the protocol quantifies arsenobetaine (AsB), iAs^III^, iAs^V^, and the 2 main arsenic metabolites observed in human urine (monomethylarsonic acid, MMA^V^ and dimethylarsinic acid, DMA^V^). In a separate study, the protocol was revised to include proteolytic enzymes to increase arsenic extraction efficiency from tuna fish reference material (Liu et al. 2015), finding that the papaya plant enzyme, papain, increased extraction efficiency from 28% to 55%.

The above protocol that combined enzymatic proteolysis and HPLC-ICPMS represents the current state-of-the-art arsenic extraction and speciation protocol for animal tissue. In this study, we applied this protocol to mouse samples and evaluated key methodologic steps, including enzymes for digestion and common tissue maceration options (Fig. S1). We then evaluated background arsenic levels in commercial lab mouse diets and tested the impacts of diet type, food restriction (fasting), and microbiome status on inter-individual variability of arsenic species in tissue (stool, liver, and bladder) in mice receiving controlled, human-relevant oral doses of iAs^III^. Finally, we evaluated the inter-lab portability of the HPLC-ICPMS technique. Our results help unify methods for arsenic speciation in murine models of human arsenic exposure and highlight the need for adequate sample sizes to better account for important inter-individual variability in arsenic exposure (i.e. body burden).

## Materials and methods

### Chemical reagents

Filter-sterilized stock solutions (10 g/l) were made for AsB (95% purity, Sigma-Aldrich, St. Louis, Missouri), arsenic (III) oxide (iAs^III^, 99.995% trace metals basis, Sigma-Aldrich, St. Louis, Missouri), sodium arsenate dibasic heptahydrate (iAs^V^, ≥98% pure, Sigma-Aldrich, St. Louis, Missouri), methylarsonic acid (MMA^V^, 95%, Wako Chemicals, Richmond, Virginia), and cacodylic acid (DMA^V^, 99%, Sigma-Aldrich, St. Louis, Missouri). Working solutions of arsenic species (0.5, 1, 5, 10, 50, and 100 µg/l) were made fresh daily by serial dilution from the stock solutions. Papain was purchased from Thermo Scientific (Waltham, Massachusetts), and pepsin was purchased from Sigma-Aldrich (St. Louis, Missouri). The mobile phase for HPLC analyses was made with HPLC-grade ammonium phosphate dibasic (Sigma-Aldrich, St. Louis, Missouri), and pH was adjusted using trace metal grade ammonium hydroxide (VWR, West Chester, Pennsylvania).

### Enzyme comparisons

Enzyme-assisted extraction methods were adapted from Liu et al. (2015), who evaluated several protease digestions of chicken breast meat, and Peng et al. (2014), who adapted previous pepsin digestion methods for chicken liver. A control papain solution (20 g/l) was made using ultrapure water (MilliQ) and incubated with shaking in a 60 °C water bath for 12 h (overnight). Similarly, a control 20 g/l pepsin solution with 0.5% HCl (trace metal grade, Fisher Scientific) was made and incubated overnight with shaking at 37 °C. To evaluate interaction of these enzymes with arsenicals, arsenic stock solutions were added to papain or pepsin solutions at a final concentration of 5 µg/l and incubated overnight as described for control solutions. To evaluate digestion of tissue, 3 replicate 0.1 g samples from the liver of an arsenic-unexposed mouse were mixed with 400 µl aliquots of pepsin solution, macerated using a mechanical tissue homogenizer (Omni International, Kennesaw, GA) at 20,000 rpm for 1 min, and incubated overnight with shaking as described above. Six replicate 0.1 g samples from the liver of an iAs^III^-exposed mouse (10 µg per g body weight) were mixed with 400 µl aliquots of papain or pepsin solutions (*n* = 3 each), macerated using a mechanical tissue homogenizer, and incubated overnight as described above. Following incubation, samples were centrifuged at 12,000 rpm for 5 min, and supernatants were filtered using a 3KDa MWCO membrane filter (MilliporeSigma, Burlington, Massachusetts) by centrifugation at 12,000 rpm for 30 min at 4 °C. Filtered samples were kept at −80 °C until HPLC-ICPMS analysis.

All 3 KDa membrane filters were washed before use to eliminate the humectant, which can interrupt arsenic signal using HPLC-ICPMS. Filters were placed in 50 ml conical tubes with 30 ml ultrapure water and shaken/incubated at room temperature for 5 min. After 3 rounds of washing, filters were stored in a new tube with ultrapure water at 4 °C.

### Papain proteomics

A sample of the commercial papain used in this work was submitted to the IDeA National Resource for Quantitative Proteomics (Little Rock, Arkansas) for proteomics analysis. The workflow involved digestion of the papain suspension with trypsin, followed by protein identification using an Orbitrap Fusion LC-MS System.

### Tissue maceration

For initial assessments of bead beating, 4 different types of lysing matrices (provided in 2 ml volume tubes) were obtained from 2 commercial vendors; lysing matrices A (garnet and ceramic beads), B (silica spheres), and E (ceramic and silica spheres and glass beads) were obtained from MP Biomedicals (Santa Ana, California, United States), and PowerBead Pro (ceramic spheres) was obtained from Qiagen (Hilden, Germany). To evaluate the different matrix materials for background arsenic, 400 µl aliquots of ultrapure water were added to each lysing matrix tube and then beaten for 1 min using the mini-BeadBeater 24 (BioSpec Products Inc., Bartlesville, Oklahoma). Tubes were centrifuged at 12,000 rpm for 5 min, and supernatant was filtered (3 KDa membrane filter) by centrifugation (12,000 rpm for 30 min at 4 °C).

Additional experiments included acid washing and boiling to remove background arsenic from the commercial beads (i.e. EPA extraction method 3050B without reflux). Briefly, 200 µl of 50% nitric acid and 500 µl of 20% hydrochloric acid were added to each lysing matrix tube followed by mixing by inversion and incubating at 95 °C for 30 min. After cooling to room temperature, the acid solution was removed, and the matrix was washed with 1 ml of ultrapure water and vortexed for 10 s. Tubes were centrifuged at 12,000 rpm for 1 min, and the water was removed. Washing with acid and/or ultrapure water was repeated 4 times, and tubes were dried in a heat block in a chemical hood at 70 °C overnight. For spiking experiments, 400 µl of ultrapure water containing 10 µg/l of each arsenic standard (AsB, iAs^III^, iAs^V^, MMA^V^, DMA^V^) was added to washed/dried tubes.

For maceration by bead beating, 0.1 g of fresh liver was mixed with 400 µl ultrapure water and transferred to an acid-washed lysing matrix tube. Tubes were beaten for 1 min, centrifuged (12,000 rpm for 5 min), and supernatant was filtered (3 KDa membrane filter) by centrifugation at 12,000 rpm for 30 min at 4 °C. Liver and stool samples from an unexposed germ-free C57BL/6 mouse and from a germ-free C57BL/6 mouse exposed to 1 µg iAs^III^ per g body weight were used for tissue comparisons. For comparing bead-beating disruption against mechanical homogenization, another 0.1 g fresh liver sample was mixed with 400 µl ultrapure water and homogenized using Omni THq Homogenizer (Omni International, Kennesaw, Georgia) at 20,000 rpm for 1 min. Samples were centrifuged (12,000 rpm for 5 min), and supernatants were collected and filtered (3 KDa membrane filters with 12,000 rpm centrifugation for 30 min at 4 °C).

### Experimental animals

All mouse experiments were conducted under protocols pre-approved by the Montana State University Institutional Animal Care and Use Committee. Experimental mice were housed at the Montana State University Animal Resource Center, an American Association for the Accreditation of Laboratory Animal Care-accredited facility. C57BL/6J mice were purchased from The Jackson Laboratory and housed in individually ventilated cages with sterilized bedding and under specific pathogen-free conditions, including protection from murine norovirus. Germ-free C57BL/6 mice were maintained in hermetically sealed, HEPA-filtered, ventilated vinyl isolators and were provided with autoclaved, reverse-osmosis water (Hellenbrand, Waunakee, Wisconsin, United States) and LabDiet mouse chow (5013 Autoclavable Rodent Breeder Diet, LabDiet, St. Louis, Missouri, United States) to ensure a sterile environment. All food and water for the germ-free mice were tested for contamination before use. Germ-free status was monitored as previously described (Coryell et al. 2018). Briefly, the germ-free status of the chow and mice stool was routinely verified using both aerobic and anaerobic culture methods on rich media (Mueller-Hinton broth and agar plates), along with periodic PCR assays targeting the bacterial 16S rRNA gene, with DNA extracted from stool samples serving as the template.

### Murine arsenic exposures

All arsenic exposures were done using iAs^III^ and age-matched (7 to 13 wk old) mice of both sexes. For enzyme digestion comparisons (described above), an arsenic-unexposed C57BL/6J mouse was humanely euthanized by isoflurane overdose, and the liver was collected. Liver tissue from this mouse was compared with the same tissue from another C57BL/6J mouse exposed to 10 mg/l iAs^III^ in drinking water ad libitum for 12 h prior. For tissue maceration comparing fasting, diet, and the microbiome, conventional and germ-free C57BL/6J mice were exposed to 1 µg iAs^III^ per g body weight via oral gavage and euthanized after 24 h. For the urine vs. bladder correlation experiment, a group of male (*n* = 5) and female (*n* = 5) mice were used. Unexposed male (*n* = 1) and female (*n* = 1) mice were randomly selected as baseline controls and euthanized. The remaining mice were exposed to 1 µg iAs^III^ per g body weight via oral gavage, and male–female pairs were randomly selected and euthanized every 3 h throughout the 12-h exposure experiment. Immediately after euthanasia, urine was collected from the bladder using a sterile syringe. In all experiments, stool was collected just prior to euthanasia, and tissues (liver and/or bladder) were collected post-euthanasia. For fasting experiments, food and water were removed for 3 h prior to iAs^III^ exposure via oral gavage (1 µg iAs^III^ per g body weight), and after an additional 3 h of fasting, food and water were reintroduced. Animals were euthanized 24 h post-gavage. For diet evaluations, mice eating a standard lab chow (5053 PicoLab Rodent Diet 20, LabDiet, St. Louis, Missouri) were switched to a purified powdered chow (110700 AIN-93G Purified Rodent Diet, Dyets, Bethlehem, Pennsylvania) for 1 wk before iAs^III^ exposure.

### Processing of mouse tissues

Unless stated otherwise, 0.1 g samples of liver and stool or 0.03 g samples of urinary bladder tissue were mixed with 400 µl of pepsin solution for digestion. Tissues were processed using a mechanical tissue homogenizer and filtration as described above.

### High-performance liquid chromatography–inductively coupled plasma mass spectrometry

HPLC was performed using an Agilent 1260 HPLC system (Agilent Technologies), which included a quaternary pump, an online degasser, and a system control unit. Arsenic species were separated using a Hamilton PRP-X100 anion-exchange column (250 × 4.1 mm, 10 µm particle size). Both samples and standard solutions were introduced via a 50 µl sample loop. Separation of arsenic compounds was achieved with isocratic elution using 10 mM (NH_4_)_2_ HPO_4_ at pH 8.25 (adjusted with trace metal grade NH_4_OH) as the mobile phase. The flow rate was maintained at 1.5 ml/min throughout the chromatographic process, with a total separation time of 10 min for the arsenic species. Identification of arsenic species was achieved using an Agilent 7800 ICPMS system and was based on matching retention times with single standards on the PRP-X100 column. The ICPMS operating conditions were managed using a daily tuning solution to ensure accuracy.

For HPLC-ICPMS comparisons between laboratories, the above setup (lab A) was compared with a different instrument setup used in another laboratory (lab B). Lab B parameters included a degasser, an HPLC column oven, an HPLC binary pump, and a standard autosampler (NexSar HPLC, PerkinElmer). This setup was connected to the ICPMS (NexION 1000, PerkinElmer) for monitoring ^75^As (m/z = 75). Two commercial columns were utilized (injection volume: 100 µl): A cation exchange column (ChromSep IonoSpher 5 C, 100 × 3.0 mm, 5 µm particle size, Agilent Technologies, Inc., Santa Clara, California) with a mobile phase consisting of 20 mM pyridine (pH 2.7, adjusted with formic acid) and a C18 reverse-phase column (BioBasic-18, 250 × 4.6 mm, 5 µm particle size, Thermo Fisher Scientific, Waltham, Massachusetts) with a mobile phase composed of 3 mM malonic acid and 5% methanol (v/v), adjusted to pH 5.95 with tetrabutylammonium hydroxide. Standard solutions containing 0, 1, 2, 5, 10, 20, and 50 µg/l of iAs^III^, iAs^V^, MMA^V^, and DMA^V^ were freshly prepared and analyzed at the beginning of each batch of sample analyses by the C18 column. For the cation exchange column assays, standard solutions containing 0, 1, 2, 5, 10, and 20 µg/l of AsB, TMAsO, and AsC were used. iAs^V^, MMA^V^, DMA^V^, AsB, TMAsO, and AsC in the samples were quantified using Clarity software (version 8.4.0.47, DataApex Ltd., Prague, Czech Republic) based on the calibration curves prepared from the results of the above-mentioned standard solutions. The peaks of iAs^III^ and AsB overlap on the C18 column due to their close retention time, whereas these 2 species are clearly separated by the cation exchange column method. Therefore, iAs^III^ was quantified by subtracting AsB levels determined by the cation exchange column method from the sum of As^III^ and AsB determined by the C18 column method. Arsenic species were eluted isocratically at a flow rate of 1 ml/min at 25 °C. Quality control measures included the analysis of reagent blanks, spiking with the arsenic species of interest, and performing duplicate readings as necessary. AsB in the samples was quantified using Clarity software (version 8.4.0.47, DataApex Ltd., Prague, Czech Republic) based on AsB standard solutions.

Both labs evaluated Standard Reference Material 2669 (Arsenic Species in Frozen Human Urine) obtained from the National Institute of Standards and Technology (NIST). This material consisted of 2 vials containing different levels of 7 arsenic species (AsB, arsenocholine, iAs^III^, iAs^V^, DMA^V^, MMA^V^, and trimethylarsine oxide). Only AsB, iAs^III^, iAs^V^, DMA^V^, and MMA^V^ could be quantified with set ups in both laboratories and included in this study (Fig. S5). For sample comparison, the same person in lab A processed liver samples from exposed mice. After processing, samples were divided and aliquoted into 2 mass spectroscopy vials. Half of the samples were shipped overnight on dry ice to lab B for analysis, and the other half were evaluated at lab A.

### Statistical analyses

All statistical analyses were performed in GraphPad Prism (Version 9.4.1, La Jolla, California). Parametric statistics were used when normality was supported by either the D’Agostino and Pearson test or the Kolmogorov–Smirnov test. For 2-way ANOVAs, data were entered with treatments in columns and arsenic species in rows such that the treatment effect on total arsenic equated to the main column factor, and treatment effects on individual arsenic species equated to the column–row interaction. When normality was not supported, data transformation was attempted (log base 10 with log(*x* + 1) used for zero imputation). If transformation proved sufficiently normal, parametric statistics were applied to the transformed data (*P*-values represent Sidak’s multiple comparison testing). If this attempt at transformation did not satisfy normality, non-parametric test equivalents were used. Mann–Whitney (total arsenic) and multiple Mann–Whitney tests with FDR correction (individual arsenic species) were used to evaluate treatment effects. Multiple Kruskal–Wallis tests with FDR correction were used for non-normal 1-way analysis. For FDR correction, the desired Q (false-positive rate) was set to 10%, and *q*-values <0.05 were considered significant and reported in figures. For clarity and ease of comparison, untransformed data are shown in all figures. Correlation analysis was used to test the relationship between 2 normal (Pearson’s *r*) or non-normal (Spearman’s *r*) variables, and simple linear (least squares) regression was used to fit a straight line to the data. An outlier was identified as noted in the text using the ROUT method (default *Q* = 0.5%) in Prism. Variance between 2 treatment groups was evaluated using an *F* test or between 3 or more groups using Levene’s median test. All statistical tests and data transformations are noted in figure legends.

## Results

### Papain- versus pepsin-assisted extraction for recovery of arsenic species from tissue

**Fig. 1.**
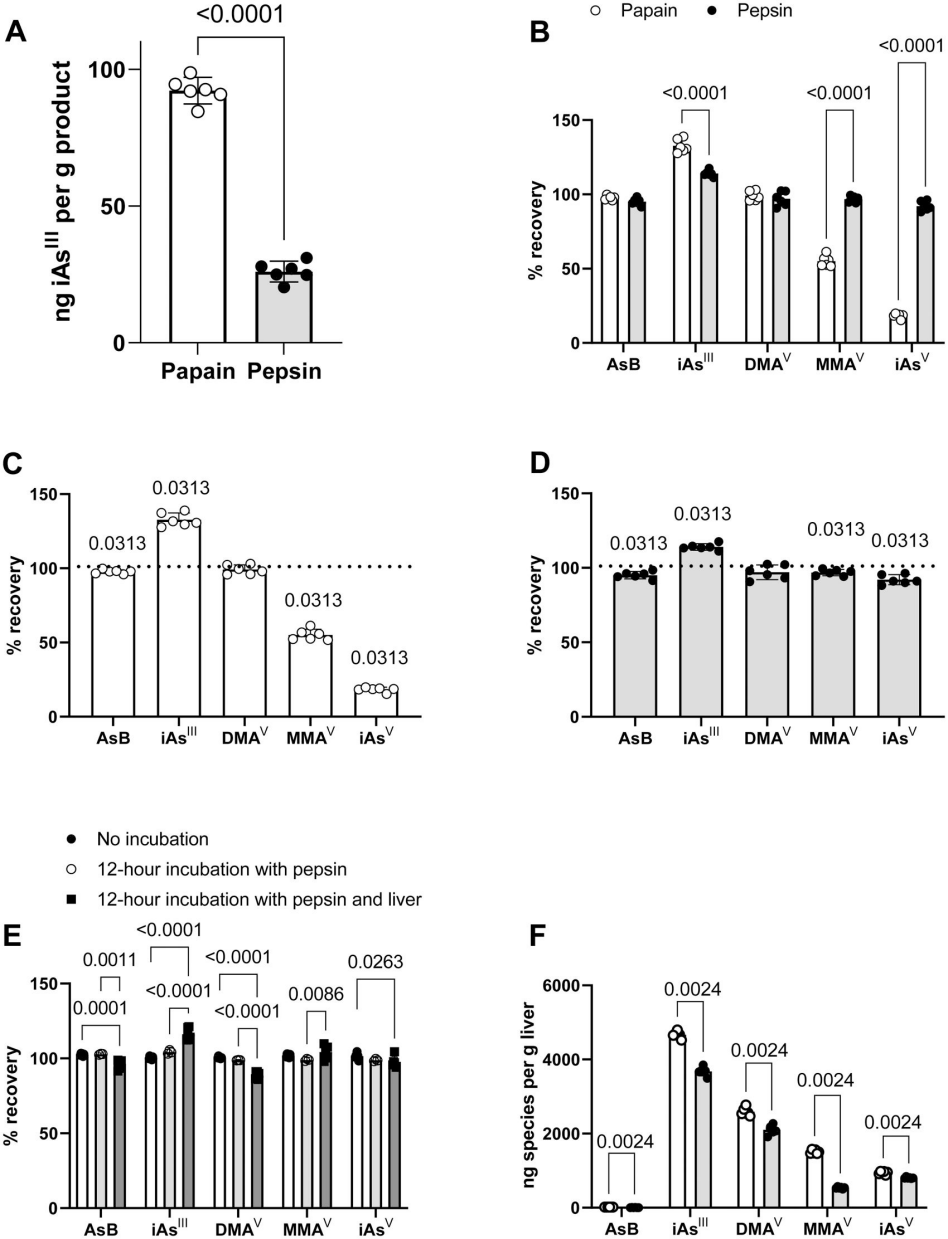
Enzyme-assisted extraction of arsenic from murine tissues using papain and pepsin. Background iAs^III^ in commercial papain and pepsin preparations (A, Welch’s *t*-test). Levels of arsenic species standards following incubation with papain and pepsin (B, 2-way ANOVA with Sidak’s test for multiple comparisons). Recovery efficiencies of papain (C) and pepsin (D) tested against the theoretical threshold of 100% (1-sample Wilcoxon test). Recovery of arsenic species standards without incubation, with 12-h incubation in pepsin alone, or with 12-h incubation with pepsin and murine liver homogenate (E, 2-way ANOVA with Tukey’s adjustment). Recovery of arsenic species from the liver of an exposed mouse using either papain- and pepsin-assisted extraction (F, multiple Mann–Whitney tests and FDR correction). Panels B, C, D, and F share legend shown in panel B.

Papain and pepsin were both shown previously to increase the efficiency of arsenic extraction from chicken breast meat (Liu et al. 2015). To evaluate these enzymes for arsenic extraction from mouse tissues, we first quantified background arsenic levels in commercial enzyme products. Individual commercial preparations of both papain and pepsin contained detectible iAs^III^, but papain contained 3.5-fold higher iAs^III^ per gram of product (Fig. 1A). No other arsenic species were observed. To evaluate enzyme-specific impacts on the recovery of arsenic species, papain and pepsin preparations were spiked with arsenic species standards (5 µg/l final concentration) and incubated overnight (12 h; see Materials and methods). Differences in recovery of iAs^III^, MMA^V^, and iAs^V^ were observed between the 2 enzymes (Fig. 1B) and when plotted and tested against a theoretical 100% recovery, papain incubation increased iAs^III^ by 31% and decreased AsB, MMA^V^, and iAs^V^ by 2%, 46% and 81%, respectively (median percentages) (Fig. 1C). Pepsin incubation was closer to 100% for all species, but still increased iAs^III^ by 14% and decreased AsB, MMA^V^, and iAs^V^ by 5%, 3%, and 9%, respectively (Fig. 1D).

Because mammalian liver contains proteases, we next evaluated whether incubation of arsenic standards with naïve murine liver homogenate (i.e. from a mouse unexposed to arsenic) influenced recovery beyond differences expected from incubation with pepsin alone or no incubation at all (Fig. 1E). No significant differences in arsenic species recovery were observed when incubated for 12 h with pepsin alone (no incubation vs. 12 h of incubation with pepsin). However, incubation with liver seemed to impact the levels of all species compared with either no incubation or incubation with pepsin alone. These differences ranged in magnitude from relatively minor for AsB (6% decrease), MMA^V^ (5% increase), and iAs^V^ (4% decrease) to larger differences observed for iAs^III^ (increased by 16%) and DMA^V^ (decreased by 12%). These results suggest that the impact of pepsin digestion on the recovery of arsenic species in the liver is less than the impact of innate hepatic enzymes.

Finally, arsenic recovery was compared in papain vs. pepsin digests of liver samples from a mouse exposed to 10 mg/l iAs^III^ ad libitum in drinking water for 12 h (see Materials and methods). Mice were given purified (MilliQ) drinking water during all exposures (here and below), and as expected from de-ionized water, no arsenic species were observed (data not shown). Consistent with published results (Liu et al. 2015), higher levels were observed for each species using papain compared with pepsin (Fig. 1F). However, the higher iAs^III^ background (Fig. 1A) and percent recovery differences following incubation with arsenic standards (Fig. 1C) suggested a greater potential to confound the level of some species, perhaps even by transformations, particularly the reduction of iAs^V^ to iAs^III^. Because the commercial papain product is a crude plant extract, we conducted a proteomics analysis to identify enzymes with the potential to convert arsenic species. Although the chymopapain protease enzyme was a dominant protein, the product also contained other enzymes (Table 1). Notably, glyceraldehyde-3-phosphate dehydrogenase was identified (100% probability), which has been documented to catalyze iAs^V^ reduction in human red blood cells, hemolysate, and rat liver cytosol (Gregus and Nemeti 2005; Nemeti et al. 2006). Collectively, these results suggest that this commercial papain product can interconvert arsenic species, thereby complicating interpretations even with increased extraction efficiency. The experiments conducted below utilized pepsin digestion exclusively.

**Table 1.** Proteins identified in the commercial papain protease product.

Identified proteins	UniProt accession number	Molecular weight	Total spectrum count	Protein identification probability
Chymopapain (fragment): OS = *Carica papaya*, OX = 3649, PE = 3, SV = 1	F6KSW9	39 kDa	286	100
Chymopapain: OS = *Carica papaya*, OX = 3649, PE = 1, SV = 2	P14080	39 kDa	270	100
Papaya proteinase 4: OS = *Carica papaya*, OX = 3649, PE = 1, SV = 3	P05994	39 kDa	74	100
Papaya proteinase omega: OS = *Carica papaya*, OX = 3649, GN = omegaii, PE = 2, SV = 1	Q42673	41 kDa	71	100
Caricain: OS = *Carica papaya*, OX = 3649, PE = 1, SV = 2	P10056	39 kDa	68	99
Glutamine cyclotransferase: OS = *Carica papaya*, OX = 3649, PE = 1, SV = 1	O81226	33 kDa	64	100
Papain: OS = *Carica papaya*, OX = 3649, PE = 1, SV = 1	P00784	39 kDa	67	100
Endochitinase: OS = *Carica papaya*, OX = 3649, PE = 1, SV = 1	P85084	27 kDa	35	100
Cysteine proteinase 3 (fragment): OS = *Vasconcellea cundinamarcensis*, OX = 35926, PE = 1, SV = 1	P32956	5 kDa	24	100
Papaya barwin-like protein: OS = *Carica papaya*, OX = 3649, PE = 1, SV = 1	U5HK42	14 kDa	13	100
Cysteine proteinase 2 (fragment): OS = *Vasconcellea cundinamarcensis*, OX = 35926, PE = 1, SV = 1	P32955	5 kDa	9	100
Latex serine proteinase inhibitor: OS = *Carica papaya*, OX = 3649, PE = 1, SV = 1	P80691	21 kDa	8	100
Cysteine proteinase inhibitor: OS = *Carica papaya*, OX = 3649, PE = 2, SV = 1	Q39561	11 kDa	8	100
GDSL esterase/lipase: OS = *Carica papaya*, OX = 3649, PE = 1, SV = 1	P86276	38 kDa	5	100
Glyceraldehyde-3-phosphate dehydrogenase (fragment): OS = *Carica papaya*, OX = 3649, GN = GAPDH, PE = 2, SV = 1	M4I2N9	29 kDa	3	100
Protein TIC 214: OS = *Jacaratia spinosa*, OX = 1222208, GN = YCF1, PE = 3, SV = 1	A0A7L8UVM6 (+1)	222 kDa	2	98
CST complex subunit CTC1 (fragment): OS = *Carica papaya*, OX = 3649, PE = 2, SV = 1	J7GLC1	45 kDa	3	92
Dicer-like 4 (fragment): OS = *Carica papaya*, OX = 3649, PE = 2, SV = 1	A0A343J651	119 kDa	2	69
Heat shock protein 70: OS = *Carica papaya*, OX = 3649, PE = 2, SV = 1	A0A1I9RH42	71 kDa	2	98
Elongation factor 1-alpha (fragment): OS = *Carica papaya*, OX = 3649, GN = EF1, PE = 2, SV = 1	M4I309	37 kDa	2	95

### Bead-beating versus mechanical tissue maceration for recovery of arsenic species

**Fig. 2.**
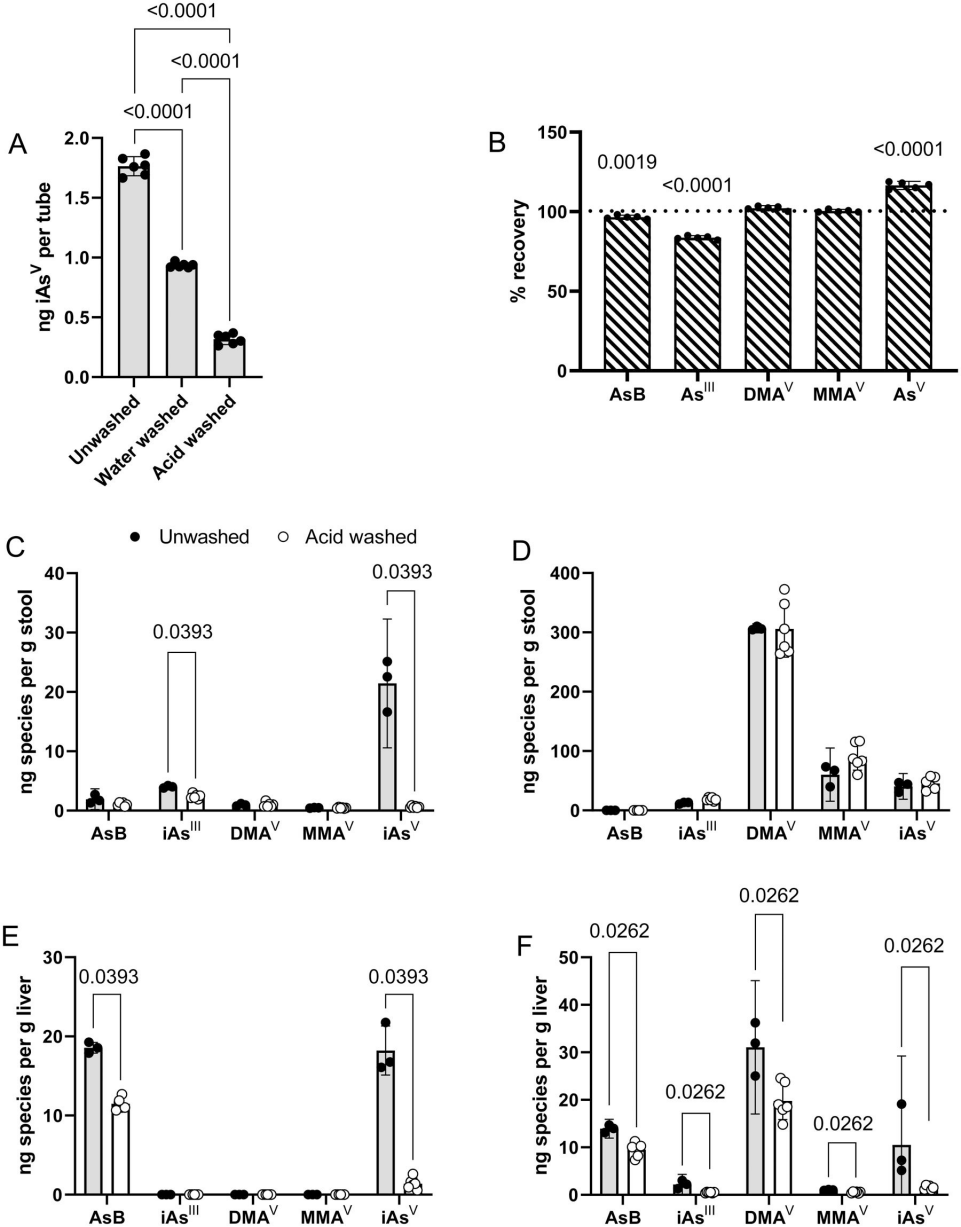
Evaluation of bead beating for murine tissue maceration and arsenic speciation. Background iAs^V^ in bead beat tubes containing lysing matrix B before and after washing with water and strong acid (A, one-way ANOVA with Tukey’s adjustment). Recovery efficiency of arsenic species standards following incubation with lysing matrix B (B, 1 sample 2-tailed *t*-test with Bonferroni adjustment). Arsenic species in stool of an unexposed (C) and exposed (D) mouse before and after acid washing of matrix B beads (multiple Mann–Whitney tests with FDR correction). Arsenic species in liver of an unexposed (E) and exposed (F) mouse before and after acid washing (multiple Mann–Whitney tests in both panels with FDR correction). Legend for unwashed and acid-washed bead beat tubes are identical for panels C and D and shown in panel C.

Two popular options for macerating medium to large numbers of tissue samples are bead beating and tube-sized tissue homogenizers. To compare arsenic extraction and speciation between these techniques, we first quantified background arsenic levels in 4 different commercial bead beat tubes containing lysing matrices. Significant levels of iAs^V^ (i.e. above our limit of detection) were detected in all 4 lysing matrix types, with the lowest level detected in lysing matrix B (Fig. S2). Pre-washing matrices with acid (see Materials and methods) removed background iAs^V^ for 2 lysing matrices, including matrix B (Fig. S2). Two batches of tubes with lysing matrix B were evaluated again using both distilled water and acid washing. Both decreased iAs^V^ background, but acid washing performed significantly better (Fig. 2A). Spiking experiments with washed lysing matrix B tubes and arsenic standards showed complete recovery of DMA^V^ and MMA^V^ and only a slight decrease (3%) of AsB (Fig. 2B). Inorganic arsenic species were more affected by bead beating with a 16% decreased recovery of iAs^III^ and 17% increased recovery of iAs^V^ (Fig. 2B).

To evaluate the effect of bead pre-washing on recovery from mouse tissue, stool and liver samples were collected and processed from an unexposed germ-free mouse (background) and a germ-free mouse 24 h after exposure to a controlled dose of 1 µg iAs^III^ per g body weight (see Materials and methods). Background iAs^III^ and iAs^V^ levels in stool were affected by acid washing the lysing matrix prior to use (Fig. 2C). However, differences in arsenic species recovery from stool samples of the exposed mouse due to matrix washing did not reach statistical significance (Fig. 2D), suggesting that the washing effect is small relative to overall stool levels. In the liver, acid-washed beads yielded significantly lower background levels of AsB and iAs^V^ (Fig. 2E) and lower levels of all species in the liver from the exposed mouse (Fig. 2F). Collectively, these results indicate that acid washing the matrix beads reduces background levels of all arsenic species, particularly iAs^V^, and lowers overall levels in the liver of exposed animals. We draw attention to the presence of AsB in liver samples from the unexposed mouse (Fig. 2E), which suggests an independent (background) exposure source; this formed the basis for additional experiments (see below).

**Fig. 3.**
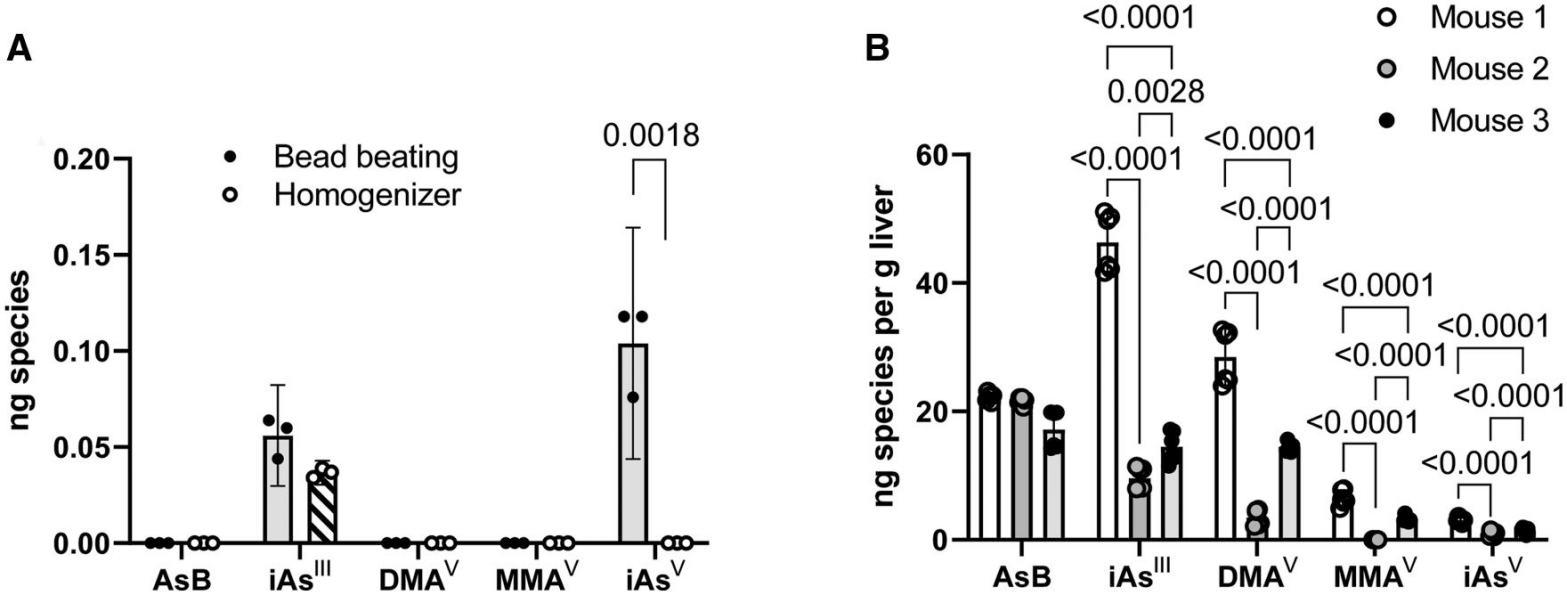
Comparison of bead beating and mechanical homogenization for murine tissue maceration to facilitate arsenic extraction. Background levels of arsenic species were compared in tubes mimicking bead beating or mechanical homogenization in the absence of tissue (A; *t* tests used to evaluate differences in iAs^III^ and iAs^V^). Combined arsenic species (i.e. levels quantified by both bead beating and homogenization) in liver samples from 3 mice following controlled exposure to iAs^III^ (B; 2-way ANOVA of log-transformed data with Tukey’s adjustment). Each bar represents the mean value of replicate liver samples from the same mouse (technical replicates of the same liver), error bars represent 95% CI of the mean, and dots represent individual values (from technical replicates). Dot shading represents individual mice (legend). Bars are shaded for ease of comparison only.

If the lysing matrix of bead beat tubes contains a significant source of inorganic arsenic species, we expected that maceration using a different approach (e.g. mechanical tissue homogenizer with pepsin digestion) would yield a lower iAs^V^ and iAs^III^ background signal. Maceration was mimicked without tissue using acid-washed bead beat tubes or in-tube, mechanical homogenization (see Materials and methods), incubated with pepsin digestion solution, and analyzed. AsB, MMA^V^, and DMA^V^ were not observed with either maceration approach (Fig. 3A). Consistent with our hypothesis and background levels quantified previously (Figs 1A and 2A), both iAs^III^ and iAs^V^ were observed using washed bead beating tubes, whereas lower levels of iAs^III^ and no iAs^V^ were observed in tubes mimicking mechanical homogenization (Fig. 3A). We next compared arsenic species levels using bead beating versus mechanical tissue homogenization of liver samples (*n* = 3 technical replicates per liver per maceration technique) from 3 conventional C57BL/6 mice 24 h after controlled exposure to iAs^III^ (oral gavage of 1 µg per g body weight). The maceration technique (i.e. bead beating vs. mechanical homogenizer) did not impact the recovery of any arsenic species, but large inter-individual differences (i.e. liver species levels between mice) were observed (Fig. S3). Because arsenic species levels did not depend on maceration technique, data from both techniques were grouped together, and inter-individual differences were compared statistically (Fig. 3B). Large differences in arsenic species were observed between mice, with the single exception of AsB, the source of which was likely food (mouse chow) that contained fish meal (see Discussion). Given the tight control of iAs^III^ dosing in these animals, these results suggest that factors other than exposure dose impact arsenic body burden. With respect to maceration type, these results also indicate that bead beating and mechanical homogenization yield comparable results. The experiments below utilized mechanical tissue homogenization exclusively (with pepsin-assisted digestion) to evaluate different factors driving inter-individual differences in arsenic body burden.

### Arsenic species levels in urine versus bladder tissue

**Fig. 4.**
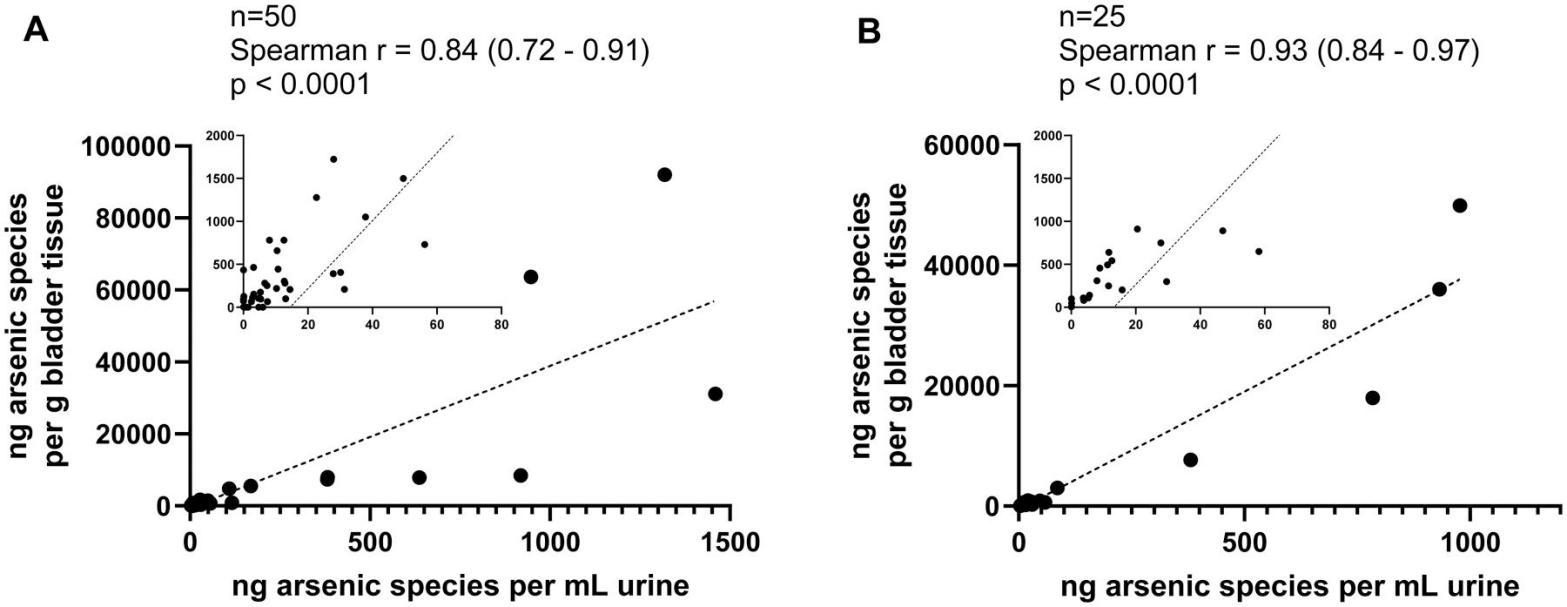
Relationship between arsenic species levels in urine and bladder tissue. Arsenic species (AsB, iAs^III^, DMA^V^, MMA^V^, and iAs^V^) were quantified in urine and bladder tissue from mice every 2 h throughout an 8-h exposure to a single oral dose of iAs^III^ (1 male and 1 female; *n* = 2 mice per time point; *n* = 10 total mice). Correlation between levels of all species was evaluated using Spearman’s rank correlation (A). Arsenic species levels were also averaged across (*n* = 2) mice at each time point to evaluate correlation strength (B). Both plots show correlation coefficients (Spearman *r*), 95% CI, and *P*-value along with an unconstrained, straight regression (dashed) line for reference. Insets in both panels are scaled to show species with lower levels (i.e. closer to the origin).

Quantification of arsenic levels in urine is a standard proxy for body burden in human epidemiology studies. Consistent urine collection from lab mice is not straightforward and may introduce variability in body burden estimates. We hypothesized that arsenic species accumulate in bladder tissue from urine, such that levels in this tissue accurately represent urine levels. To test this hypothesis, urine and bladder tissue samples were collected from a group of mice (*n* = 5 male and *n* = 5 female) throughout a 12-h exposure, ensuring a range of arsenic levels was observed. At the outset of the experiment, an unexposed male and female mouse were randomly selected and served as baseline (background) controls. All other mice were exposed to 1 µg iAs^III^ per g body weight via oral gavage, and male–female pairs were randomly selected and euthanized every 3 h (*n* = 10 mice total; pairs sampled at 0, 3, 6, 9, and 12 h). For analysis, arsenic levels in urine samples were considered the predictor of levels in bladder tissue. Urinary levels of individual arsenic species were significantly correlated with levels in bladder tissue, with the single exception of AsB, which showed a strong trend (*P* = 0.067; Fig. S4). When levels of all species in urine were plotted against levels in bladder tissue, a strong (coefficient = 0.84), significant (*P* < 0.0001) relationship was observed (Fig. 4A). Similar to livers from exposed mice (Fig. 3B), large differences were observed in urine and/or bladder samples between mice (*n* = 2) evaluated at the same time point, again suggesting inter-individual differences were a significant source of variation. To evaluate this statistically, we tested whether averaging values across mice (*n* = 2) at each time point made the relationship between urine and bladder tissue stronger (Fig. 4B). Consistent with this hypothesis, the strength of the correlation coefficient using averaged values increased to 0.93, whereas the width of the 95% CI around the coefficient decreased by 32%. Overall, these results provide strong evidence that arsenic species levels in urine are highly correlated with those in bladder tissue and that inter-individuality between mice is, again, an important source of variation.

### The impact of standard versus purified diets on arsenic burden and inter-individual variability

**Fig. 5.**
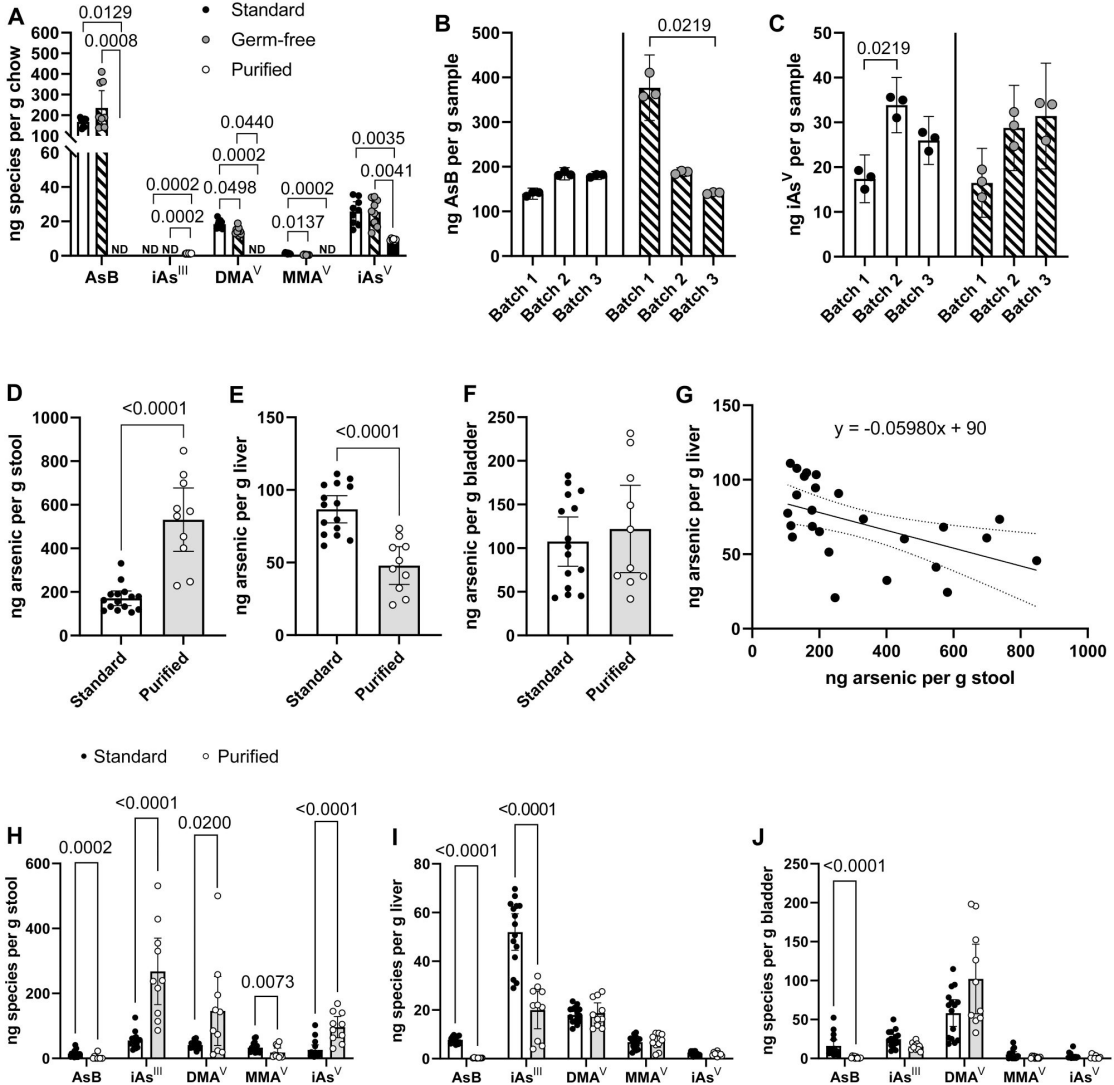
Dietary impact on arsenic body burden. Background levels of arsenic species in 3 commercial mouse diets (A; multiple Kruskal–Wallis tests with FDR correction). Background levels of AsB (B) and iAs^V^ (C) in different batches of the standard and germ-free mouse diets (Kruskal–Wallis test with Dunn’s adjustment). Total arsenic levels in stool (D), liver (E), and bladder (F) of iAs^III^-exposed mice eating either a standard or purified diet (Welch’s *t*-test of log-transformed stool and liver data). Relationship between total stool and liver arsenic (G). Unconstrained linear regression line (solid line) shown with 95% CI (dotted lines). Equation of regression line, 95% CI of slope, and goodness of fit (*R*^2^) also shown. Arsenic species in stool (H), liver (I), and bladder (J) of iAs^III^-exposed mice eating either a standard or purified diet (multiple Mann–Whitney tests with FDR correction for stool and liver; 2-way ANOVA of log-transformed bladder data with Sidak’s test for multiple comparisons).

Human studies have suggested that arsenic exposure through the diet explains at least some inter-individual variability in arsenic body burden and subsequent disease risk (Lovreglio et al. 2012; Cubadda et al. 2017), but few studies have evaluated the influence of different diets on arsenic body burden. To address this, we quantified background levels of arsenic species in 3 commercial lab mouse diets, including samples from different batches, or shipments, from the same vendors. The standard lab mouse diet used in our facility contained considerable levels of AsB, DMA^V^, and iAs^V^, as did a nutritionally equivalent, autoclavable diet used for germ-free mouse husbandry (Fig. 5A). In contrast, a “purified” diet commonly used for controlled nutrition studies had undetectable levels of AsB, DMA^V^, MMA^V^, and significantly lower levels of iAs^V^ (Fig. 5A). All 3 diets contained undetectable or trace amounts (<2.0 ng per g sample) of iAs^III^. Significant batch-to-batch variability was observed for AsB in the germ-free diet (Fig. 5B) and iAs^V^ in the standard diet (Fig. 5C), suggesting that levels also differ between manufactured batches of these diets (DMA^V^ levels were not significantly different between batches of either diet).

Controlled iAs^III^ exposures were conducted in groups of mice eating either standard or purified diets, and arsenic levels were quantified in stool, liver, and bladder samples after 24 h. Total arsenic levels (i.e. the sum of all species) differed in the stool (Fig. 5D) and liver (Fig. 5E) according to diet, whereas no difference was observed in the bladder (Fig. 5F). Mice eating the purified diet excreted more arsenic in stool and accumulated less in the liver compared with mice eating the standard diet. Interestingly, total arsenic levels in the stool and liver were correlated (Pearson’s *r* = −0.5187, *R*^2^ = 0.2690, *P* = 0.0079) on an individual mouse basis. Linear regression suggested that for every 1% increase in total stool arsenic, total arsenic in liver samples decreased by 0.06% (Fig. 5G).

With respect to individual arsenic species, AsB levels were significantly lower or undetectable in mice eating the purified diet, regardless of sample type (stool, liver, and bladder; Fig. 5H to J). In the stool and bladder, AsB levels in mice eating the purified diet did not differ significantly from the estimated limit of detection (1-sample Wilcoxon tests), and only trace amounts of AsB were detected in the liver of these animals (range = 0.17 to 0.48 ng per g tissue; LOD = 0.16 ng per g tissue). In addition, mice eating the purified diet excreted more iAs^III^, DMA^V^, and iAs^V^ and less MMA^V^ in stool (Fig. 5H) and accumulated less iAs^III^ in the liver (Fig. 5I) compared with mice eating the standard diet.

Regarding inter-individual variability, the variance in total stool arsenic was greater in mice eating the purified diet (Table 2), whereas the variance in total liver and bladder arsenic did not differ significantly. At the individual species level, the variance of both DMA^V^ and iAs^V^ was greater in the stool of mice eating the purified diet, but no other differences were statistically significant (Table 2). As noted in Table 2, AsB levels in the stool of mice eating the purified diet were at or below our detection limit, and so variance was not evaluated for this arsenical. Collectively, these results suggest that the purified diet decreased arsenic body burden but did not decrease inter-individual variability compared with the standard diet.

**Table 2.** The effect of tested factors on the inter-individuality of arsenic (total) and of HPLC-ICPMS-resolved species.

Total As (*F* test)						
**Diet (standard vs. purified)**	***F***	**DFn**	**Dfd**	***P*-value**	**Greater variance**	
Stool	11.24	9	14	0.0001	Purified diet	
Liver	1.157	9	14	0.7788		
Bladder	1.875	9	14	0.2816		
**Fasting (unfasted vs. fasted)**						
Stool	1.156	19	14	0.7541		
Liver	2.087	19	14	0.1649		
Bladder	18.56	19	14	<0.0001	Fasted	
**Microbiome (conventional vs. germ-free)**						
Stool	7.197	14	7	0.0139	Conventional	
Liver	2.006	14	7	0.2536		
Bladder	29.83	14	7	<0.0001	Germ-free	
**Arsenic species (Levene’s test)**						
**Diet (standard vs. purified)**	**Test^a^**	**Mean difference^b^**	**Adjusted *P*-value**	**Greater variance**		
**Stool**						
AsB			NA^c^			
iAs^III^	Kruskal–Wallis	−31.570	0.1641			
DMA^V^	Kruskal–Wallis	−55.370	0.0009	Purified		
MMA^V^	Kruskal–Wallis	12.870	>0.9999			
iAs^V^	Kruskal–Wallis	−41.070	0.0275	Purified		
**Liver**						
AsB			NA^c^			
iAs^III^	Kruskal–Wallis	16.870	>0.9999			
DMA^V^	Kruskal–Wallis	−10.030	>0.9999			
MMA^V^	Kruskal–Wallis	−6.467	>0.9999			
iAs^V^	Kruskal–Wallis	−0.033	>0.9999			
**Bladder**						
AsB			NA^c^			
iAs^III^	Kruskal–Wallis	14.630	>0.9999			
DMA^V^	Kruskal–Wallis	−23.770	0.5402			
MMA^V^	Kruskal–Wallis	20.270	0.8528			
iAs^V^	Kruskal–Wallis	−6.900	>0.9999			
**Fasting (unfasted vs. fasted)**	**Test^a^**	**Mean difference^b^**	**Adjusted *P*-value**	**Greater variance**		
**Stool**						
AsB	Kruskal–Wallis	−5.150	>0.9999			
iAs^III^	Kruskal–Wallis	2.450	>0.9999			
DMA^V^	Kruskal–Wallis	2.183	>0.9999			
MMA^V^	Kruskal–Wallis	28.530	0.4958			
iAs^V^	Kruskal–Wallis	−1.533	>0.9999			
**Liver**						
AsB	Kruskal–Wallis	−9.267	>0.9999			
iAs^III^	Kruskal–Wallis	9.842	>0.9999			
DMA^V^	Kruskal–Wallis	1.925	>0.9999			
MMA^V^	Kruskal–Wallis	8.558	>0.9999			
iAs^V^	Kruskal–Wallis	−10.010	>0.9999			
**Bladder**						
AsB	Kruskal–Wallis	−35.940	0.1890			
iAs^III^	Kruskal–Wallis	5.175	>0.9999			
DMA^V^	Kruskal–Wallis	−37.350	0.1545			
MMA^V^	Kruskal–Wallis	10.300	>0.9999			
iAs^V^	Kruskal–Wallis	−17.380	>0.9999			
**Microbiome (conventional vs. germ-free)**	**Test^a^**	**Mean difference^b^**	**Adjusted *P*-value**	**Greater variance**		
**Stool**						
AsB	Kruskal–Wallis	48.080	0.0049	Conventional		
iAs^III^	Kruskal–Wallis	44.650	0.0111	Conventional		
DMA^V^	Kruskal–Wallis	−2.200	>0.9999			
MMA^V^	Kruskal–Wallis	37.790	0.0481	Conventional		
iAs^V^	Kruskal–Wallis	38.810	0.0392	Conventional		
**Liver**						
AsB	One-way ANOVA	−8.035	0.0002	Germ-free		
iAs^III^	One-way ANOVA	−6.202	0.0053	Germ-free		
DMA^V^	One-way ANOVA	−0.664	0.9983			
MMA^V^	One-way ANOVA	0.346	>0.9999			
iAs^V^	One-way ANOVA	−0.203	>0.9999			
**Bladder**						
AsB	Kruskal–Wallis	−35.940	0.0688			
iAs^III^	Kruskal–Wallis	0.700	>0.9999			
DMA^V^	Kruskal–Wallis	−25.640	0.3942			
MMA^V^	Kruskal–Wallis	34.740	0.0863			
iAs^V^	Kruskal–Wallis	5.058	>0.9999			

a Kruskal–Wallis, nonparametric Kruskal–Wallis with Dunn’s multiple comparisons test; one-way ANOVA, ordinary one-way ANOVA with Sidak’s multiple comparisons test.

b Mean difference for Kruskal–Wallis is the mean rank difference.

c Observed levels were at or below the detection limit and not statistically evaluated.

### The impact of fasting on arsenic burden and inter-individual variability

**Fig. 6.**
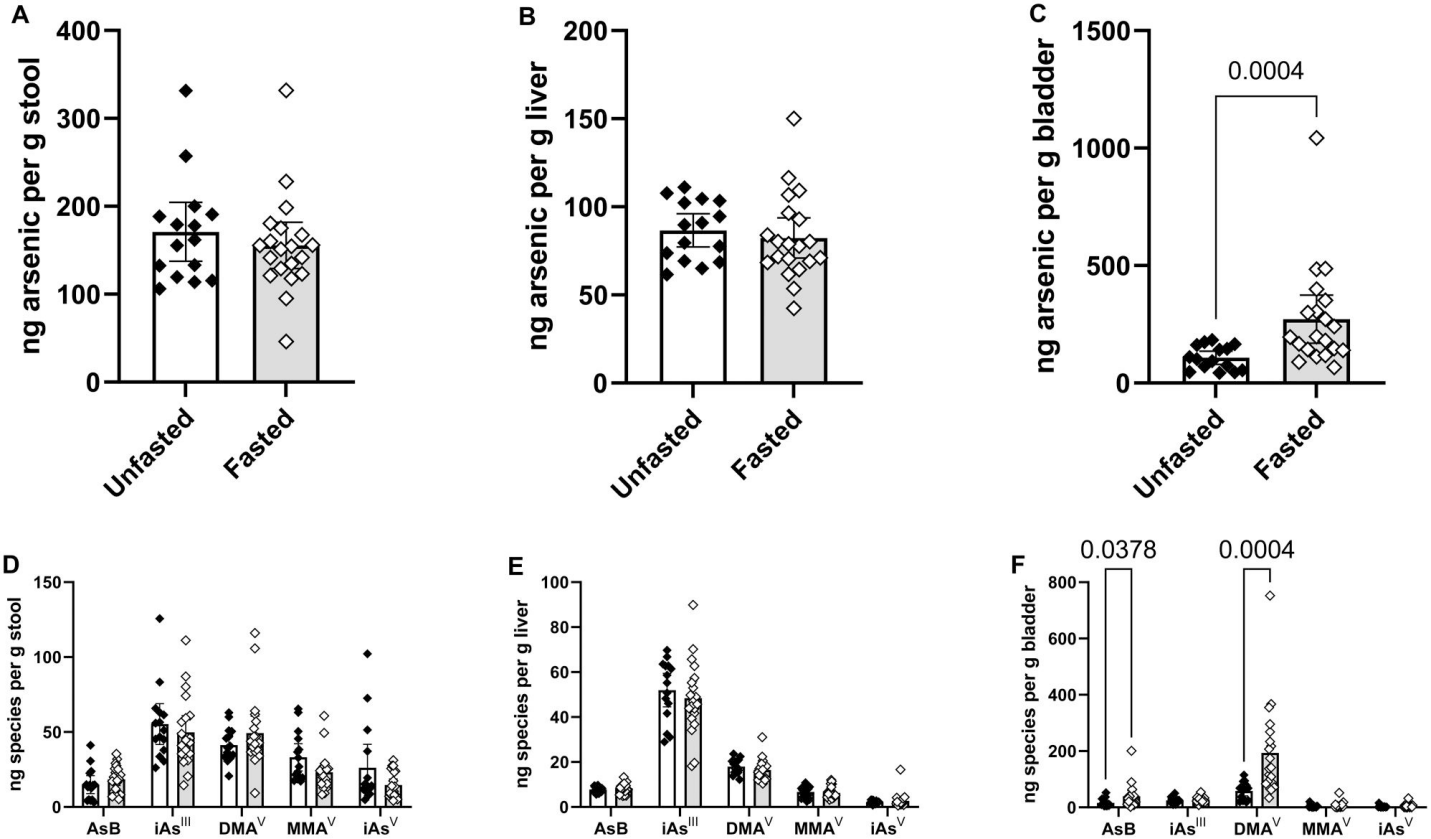
The impact of fasting on arsenic body burden. Total arsenic in stool (A), liver (B), and bladder (C) in unfasted and fasted mice (column factor from 2-way ANOVA of log-transformed data for each panel). Arsenic species in stool (D), liver (E), and bladder (F) in unfasted and fasted mice (2-way ANOVA of log-transformed data for each panel with Sidak’s test for multiple comparisons). Bars/symbols in panels D to F represent groups labeled in A to C (open bars/closed diamonds = unfasted mice, shaded bars/open diamonds = fasted mice). Bars in all plots represent group means and error bars represent 95% confidence limits.

Mice in the above experiments had constant access to food and water, and we hypothesized that the timing of arsenic exposure after eating and/or drinking may have been a significant source of the inter-individual variability. To test this hypothesis, we compared arsenic levels between exposed mice that ate a conventional diet and drank ad libitum versus groups of exposed mice undergoing food and water restriction (i.e. fasting). For the fasted group, food and water were removed for 3 hours prior to exposure and withheld for an additional 3 h following exposure. This amount of fasting had no effect on total arsenic in the stool (Fig. 6A) or liver (Fig. 6B) but increased total arsenic in the bladder (Fig. 6C). Likewise, fasting had no effect on the level of individual arsenic species in the stool (Fig. 6D) or liver (Fig. 6E) but increased both AsB and DMA^V^ in the bladder (Fig. 6F). With respect to variance, bladder samples from fasted mice exhibited greater variance in total arsenic, but otherwise, fasting had no influence on the variance of individual arsenic species (Table 2). Collectively, fasting increased the burden and inter-individual variability of total arsenic but only in the bladder.

### The impact of microbiome status (germ-free vs. conventional) on arsenic burden and inter-individual variability

**Fig. 7.**
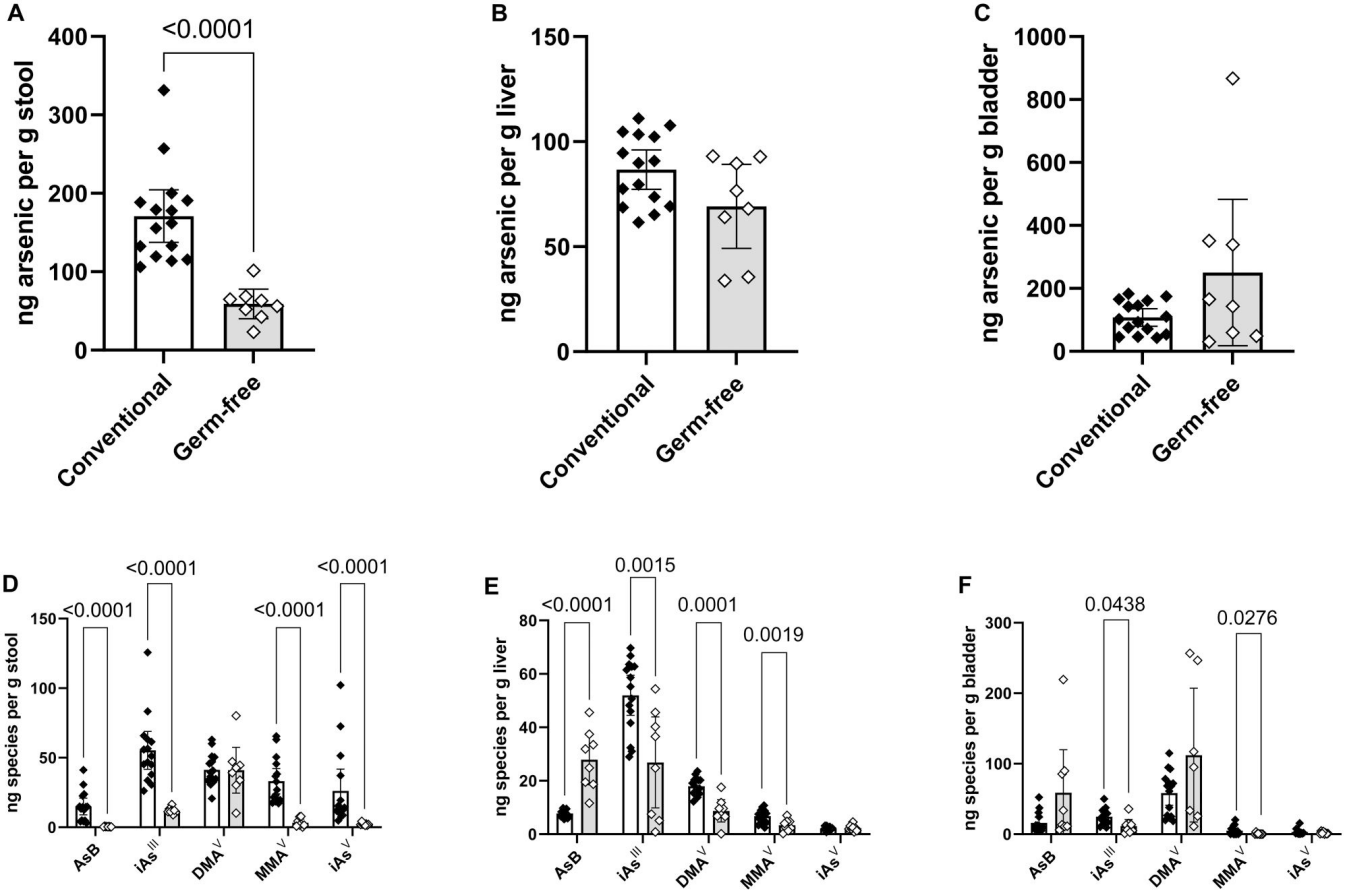
The impact of microbiome status on arsenic body burden. Total arsenic in stool (A), liver (B), and bladder (C) of conventional and germ-free mice (column factor from 2-way ANOVA of log-transformed data for stool and bladder; Welch’s *t*-test of log-transformed data for liver). Arsenic species in stool (D), liver (E), and bladder (F) in conventional and germ-free mice (2-way ANOVA of log-transformed data for stool and bladder with Sidak’s test for multiple comparisons; multiple Mann–Whitney tests for liver data with FDR correction). Bars/symbols in panels D to F represent groups labeled in A to C (open bars/closed diamonds = conventional mice, shaded bars/open diamonds = germ-free mice). Bars in all plots represent group means and error bars represent 95% confidence limits.

The quickly evolving literature documents how the gut microbiome is important for normal arsenic excretion, metabolism, and disease risk (Coryell et al. 2018; McDermott et al. 2020; Yang et al. 2021). To evaluate the microbiome’s impact on arsenic species levels, groups of conventional and germ-free mice were exposed to iAs^III^ and evaluated for species levels in the stool, liver, and bladder. Germ-free mice excreted less total arsenic in stool (Fig. 7A), but differences in total arsenic in liver (Fig. 7B) and bladder (Fig. 7C) did not reach significance. Stool levels of all species except DMA^V^ were lower in germ-free compared with conventional mice (Fig. 7D). Liver AsB levels were greater, and iAs^III^ and DMA^V^ levels were lower in germ-free compared with conventional mice (Fig. 7E). In the bladder, iAs^III^ and MMA^V^ levels were lower in germ-free compared with conventional mice (Fig. 7F). The presence of a microbiome had different effects on total arsenic variance according to sample type. Greater variance was observed in total stool arsenic of conventional versus germ-free mice; no significant difference was observed in liver samples, and less variance was observed in total bladder arsenic of conventional versus germ-free mice (Table 2). Greater variance was observed for all arsenic species in the stool of conventional versus germ-free mice except for DMA^V^ (Table 2). Liver samples from germ-free mice exhibited greater variance in AsB and iAs^III^ compared with conventional mice, and no significant differences were observed in bladder samples (Table 2). Collectively, our results indicate that the microbiome (or lack thereof) significantly influences arsenic body burden and inter-individuality of arsenic species following exposure, and the direction and extent of this influence may be tissue and arsenic species-dependent.

### Inter-lab comparison of HPLC-ICPMS-resolved arsenic species

**Fig. 8.**
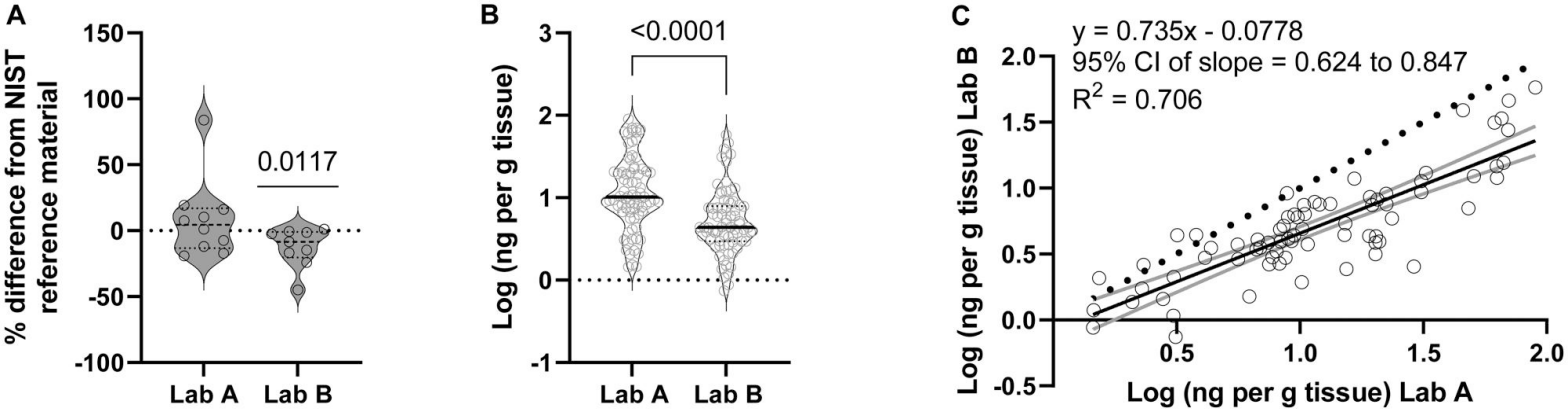
Inter-lab comparison of HPLC-ICPMS for arsenic speciation. Absolute abundance of arsenic species (AsB, iAs^III^, DMA^V^, MMA^V^, and iAs^V^) were quantified from NIST reference material (Standard Reference Material 2669) in on 2 different instruments (lab A and lab B). An outlier was detected/removed the lab B dataset. Results are shown as the percent (%) difference from values in reference material and were compared with a value of 0% difference (A, 1-sample Wilcoxon test). Liver samples from 15 iAsIII-exposed mice were evaluated by both labs (B, unpaired *t*-test of log-transformed data). The relationship between arsenic species levels quantified in labs A and B (log-transformed data) was evaluated using linear regression (C). A constrained regression line (dotted line; *y*-intercept = 0, slope = 1) was plotted for reference along with the unconstrained regression (solid black line) and 95% CI (light gray lines), equation, and goodness of fit (*R*^2^).

HPLC-ICPMS is a sensitive, accurate, precise, and widely used method for arsenic speciation, but technical differences in instrument set-up (mobile phases, chromatography columns, and instruments) may introduce variability in results. To evaluate the potential impacts of different HPLC-ICPMS-based analyses, 2 laboratories (lab A and lab B) with different instruments, instrument configurations, and buffers (see Materials and methods) quantified arsenic species in Standard Reference Material of frozen human urine from the NIST (Fig. S5). This material comes in 2 vials containing different levels (level I and level II) of 7 arsenic species, of which 5 species were measured (AsB, iAs^III^, DMA^V^, MMA^V^, and iAs^V^). Each lab prepared their own standards, and calculated results as the percent (%) difference from NIST reported values (e.g. a 0% difference = exact match, a positive % difference = lab overestimate, a negative % difference = lab underestimate). An outlier was identified in the dataset from lab B and was removed. Values from lab A were not statistically different from 0% but those from lab B underestimated NIST arsenic species levels by an average of 8% (Fig. 8A). Next, a single technician processed and prepared 15 liver samples from arsenic-exposed mice, splitting them into 2 identical aliquots prior to freezing. One aliquot of each sample was thawed and analyzed by lab A, and the matching aliquots (shipped on dry ice) were thawed and analyzed by lab B (i.e. 5 arsenic species were quantified in 15 liver samples by each lab; *n* = 75 results for each lab). A lab-specific difference was observed with levels reported by lab B being 33% lower on average compared with those reported by lab A (Fig. 8B). The lower estimate from lab B was consistent across all arsenic species, and both labs observed similar inter-individual variability in species levels (coefficient of variation; Fig. S6). Despite differences in absolute levels, individual arsenic species were significantly correlated between labs (Pearson’s *r* = 0.840, *R*^2^ = 0.71, *P* < 0.0001) and significantly linearly related (Fig. 8B). These results provide strong evidence that absolute estimates of HPLC-ICPMS-generated arsenic species levels will differ between labs due to typical, lab-specific differences in analytical instruments, configuration, and run conditions, but that relative abundances should be portable between labs.

## Discussion

Lab animal models of human arsenic exposure are useful for identifying and evaluating important factors influencing disease risk (Cohen et al. 2013; Tsuji et al. 2019). Many potentially important but complex factors, such as genetics (Xenakis et al. 2022), can be controlled in lab animals, which helps disentangle their effects. But, even under experimentally controlled conditions, our results indicate that lab mice display inter-individual responses that often go unaccounted for in scientific studies. The source of this variability is likely due to both technical and biological factors, many of which have not been evaluated previously.

### Technical sources of variability

Extraction of arsenic from animal tissue is improved with protease treatment (Peng et al. 2014; Liu et al. 2015). Papain is a crude plant extract and led to lower-than-expected levels of iAs^V^ and MMA^V^ and higher background levels of iAs^III^ compared with pepsin. Proteomics did not identify known (de)methylation enzymes that could explain the observed difference in MMA^V^, but the observed glyceraldehyde-3-phosphate dehydrogenase could have reduced iAs^V^ (Gregus and Nemeti 2005; Nemeti et al. 2006). Also, papain is a cysteine (Cys) protease with a free Cys residue in the catalytic site (Grzonka et al. 2007), whereas pepsin is an aspartic protease (Tang et al. 1973). Given that MMA^V^ is nonenzymatically conjugated with Cys at higher Cys/MMA^V^ molar ratios (≥3) and reduced to MMA^III^ (Murato et al. 2006; Sakurai et al. 2006), the free Cys in papain may have reduced both As^V^ and MMA^V^ (the papain concentration used was >10 times higher than that of arsenic). Finally, observed decreases in iAs^V^ and MMA^V^ were not fully explained by a concomitant increase in iAs^III^, suggesting that some protein-bound arsenic was preferentially removed during processing (filtration). With respect to biotransformation, lengthy (overnight) incubations of tissues invite arsenic species transformation due to reactive enzymes (e.g. AS3MT), leading to altered levels and misinterpretations. These in vitro transformations may be limited due to degradation by the digestive enzyme (e.g. pepsin), but still important.

Extraction of arsenic from animal tissue requires maceration, but there is little consensus on methodology. Mechanical (blade and/or pestle grinding) maceration of fresh or freeze-dried tissues is a common method for processing tissue from animal models and humans (Peng et al. 2014; Liu et al. 2015; Douillet et al. 2023). However, the small size of mouse tissue limits the use of certain instruments like commercial food blenders (e.g. KitchenAid; Peng et al. 2014; Liu et al. 2015). Mortar–pestle grinding of large batches of samples can be time-consuming and more prone to human error. The use of a small mechanical homogenizer and bead beating generated comparable results, though mechanical homogenization introduced lower background arsenic levels compared with bead-beating, which may not be ideal when evaluating low-level exposures (e.g. ppb range). Follow-up experiments determined that the altered concentrations of iAs^III^ and iAs^V^ in the iAs^III^ spike-recovery experiments (Fig. 2B) derived from the release of pre-existing, bead-associated iAs^V^ and surface exchange of iAs^III^ for iAs^V^ on the lysing matrix (see Supplementary Information).

Finally, we showed that labs using different HPLC-ICPMS instruments and set-ups can generate well-correlated but different absolute results (up to 30% different). Importantly, similar inter-individual variability (coefficient of variation) was observed by both laboratories, supporting large mouse-to-mouse differences. These results emphasize the point that comparisons across studies using different set-ups should focus on relative rather than absolute measures of arsenic species. These results also emphasize that biological factors, as opposed to differences in HPLC-ICPMS, likely impact inter-individual variability in arsenic species levels.

### Biological sources of variability

Urinalysis is the most common approach for estimating arsenic exposure in humans. Urine is a non-invasive specimen, but only a proxy for levels in tissues where arsenic-related pathology (e.g. cancer) develops. Mice do not urinate consistently, and engineered devices for capture (e.g. metabolic chambers) have drawbacks (see Supplementary Information). We found arsenic species levels in urine correlated well with those in the bladder during short (8-h) exposures. Our data, however, were not normal, and so this correlation (Spearman’s rank correlation) applies to ranked values only. Whether additional data might result in normality remains to be seen, but averaging values across mice significantly improved the urine–bladder correlation. In humans, detection of analytes in urine can be highly variable (>50% for some analytes) due to natural biological variability (differences in host hydration status, frequency, and volumes of urination) (Shihabi et al. 2001), so it seems reasonable that at least some of the variability observed between mice was due to similar factors. Taken together, it is important to power experiments to account for this type of variability.

This study provides evidence that diet composition, fasting, and gut microbiomes are key factors influencing the burden and inter-individuality of arsenic species in the body. Different levels of background arsenic have been observed in mouse diets (Murko et al. 2018), and our results are consistent with these findings. Results to date provide strong evidence that purified mouse diets, like AIN-93G, contain lower arsenic background compared with non-purified diets, and this is especially true for AsB. Significant AsB in non-purified mouse diets is likely derived from fish (as fish meal) being a principal source of protein (Shiomi et al. 1995; Larsen and Francesconi 2003). This is important, as in vitro studies suggest that this AsB can be transformed to other species (e.g. iAs) (Men et al. 2022; Liu et al. 2023).

We are unaware of other studies that quantified arsenic species in arsenic-exposed mice eating different diets. Studies in rats evaluated arsenic-related outcomes in reproductive tissues (testes and spermatozoa of males; uterine epithelium and ovarian cells of females), whereas eating isocaloric diets differing in protein content (18% vs. 32%) and source (whole wheat flour + chickpea flour + casein vs. pea + casein) (Mondal et al. 2013; Biswas et al. 2019; Biswas and Kumar Mukhopadhyay 2020; Biswas et al. 2023). Rats in these studies were exposed to arsenic trioxide, and only total arsenic (quantified by hydride generation atomic absorption spectrometry, HG-AAS) was reported in 1 study evaluating testes (Biswas et al. 2023), making direct comparisons difficult. That said, testicular arsenic levels were lower in rats eating the higher protein diet (32%, pea + casein), which is consistent with increased arsenic elimination via stool due to protein adsorption in the gut (Wang et al. 2021). Regardless, the total protein content of the standard (21%) and purified (20%) mouse diets examined here was almost identical, so this does not explain the higher level of arsenic excretion in stool observed in mice eating the purified diet. With respect to protein source, the purified diet contained casein supplemented with 0.3% L-cystine (the oxidized form of Cys), whereas the standard diet contained a variety of protein sources, including fish meal, soy, wheat, and whey. Currently, it is unclear whether these protein sources differ significantly in Cys content and whether this may explain observed results (see Supplementary Information).

Fasting experiments were performed to test the hypothesis that observed variability in arsenic body burden is due to eating and drinking (both the amount ingested and time since ingesting). Of the 3 factors examined, fasting had the smallest influence on arsenic body burden, affecting only 2 of the 5 species and only in the bladder. Fasting was also the only factor that did not significantly influence inter-individual variability. From this, we infer that fasting of mice had a minor impact relative to diet and microbiome. The length of fasting (3 h) was selected based on metabolite (e.g. glucose) normalization in C57BL/6J mice (Moro and Magnan 2025), and it is possible that an increased state of starvation (i.e. fasting >4 h) would yield different outcomes (see Supplementary Information).

Previous studies by our group (Coryell et al. 2018; Roggenbeck et al. 2021; Wang et al. 2021) and others (Lu et al. 2013, 2014a, 2014b; Chi et al. 2019) have shown that the gut microbiome is an important factor in arsenic exposure, and several reviews highlight potentially important mechanisms (Coryell et al. 2019; McDermott et al. 2020; Yang et al. 2021). Results reported here add to this growing body of evidence and are the first to directly compare arsenic species in tissues from conventional versus germ-free mice. The microbiome had a large impact on arsenic excretion in stool and accumulation in tissues, which is consistent with our previous findings (Coryell et al. 2018; Wang et al. 2021). The presence of a microbiome also increased inter-individuality (variance) of arsenic in stool, which further supports the hypothesis that individual-to-individual differences in this complex assemblage of microorganisms influence elimination and metabolism. The absence of a microbiome increased inter-individuality of arsenic levels in the liver but did not seem to affect those in the bladder. The overall (stool–liver–bladder) pattern suggests the microbiome’s role is strongest early on during exposures, helping regulate absorption, liver exposure, and enterohepatic circulation, all of which is consistent with a “facilitation by excretion” concept.

### Inter-individual variability and sample size

Mice, like people, naturally differ in arsenic body burden. Based on the variance observed here, many arsenic studies using mice seem inadequate with respect to sample size and design. This is important because conclusions from underpowered studies may change if/when repeated. Based only on the variance observed between conventional mice eating a standard diet, a coefficient of variation between 18% and 114% should be expected in the levels of individual arsenic species in tissues (stool, liver, and bladder). Using standard statistical assumptions (alpha = 0.05 and power = 0.8), the sample size will depend on the effect size one wishes to detect (e.g. difference between treatments). In our experience, an effect size >15% requires more than 5 mice per group, but more importantly, results should be based on more than 1 group to adequately account for cage effects. At a minimum, we suggest 2 cages of mice containing 4 to 5 mice each per treatment. These are general estimates, and studies should consider pilot studies to evaluate statistical power beforehand.

## Supplementary Material

kfag055_Supplementary_Data
