# Supplementary material for: Technical and biological factors driving inter-individual body burden of arsenic species in murine models of human arsenic exposure
Source: Toxicol Sci. 2026 May 19;209(6):kfag055. doi: 10.1093/toxsci/kfag055 (PMC13265383; doi:10.1093/toxsci/kfag055)
Supplement: kfag055_Supplementary_Data [file kfag055_supplementary_data.docx]

**Supplemental Figure 1.** Overview of study and identification of technical and biological factors examined.


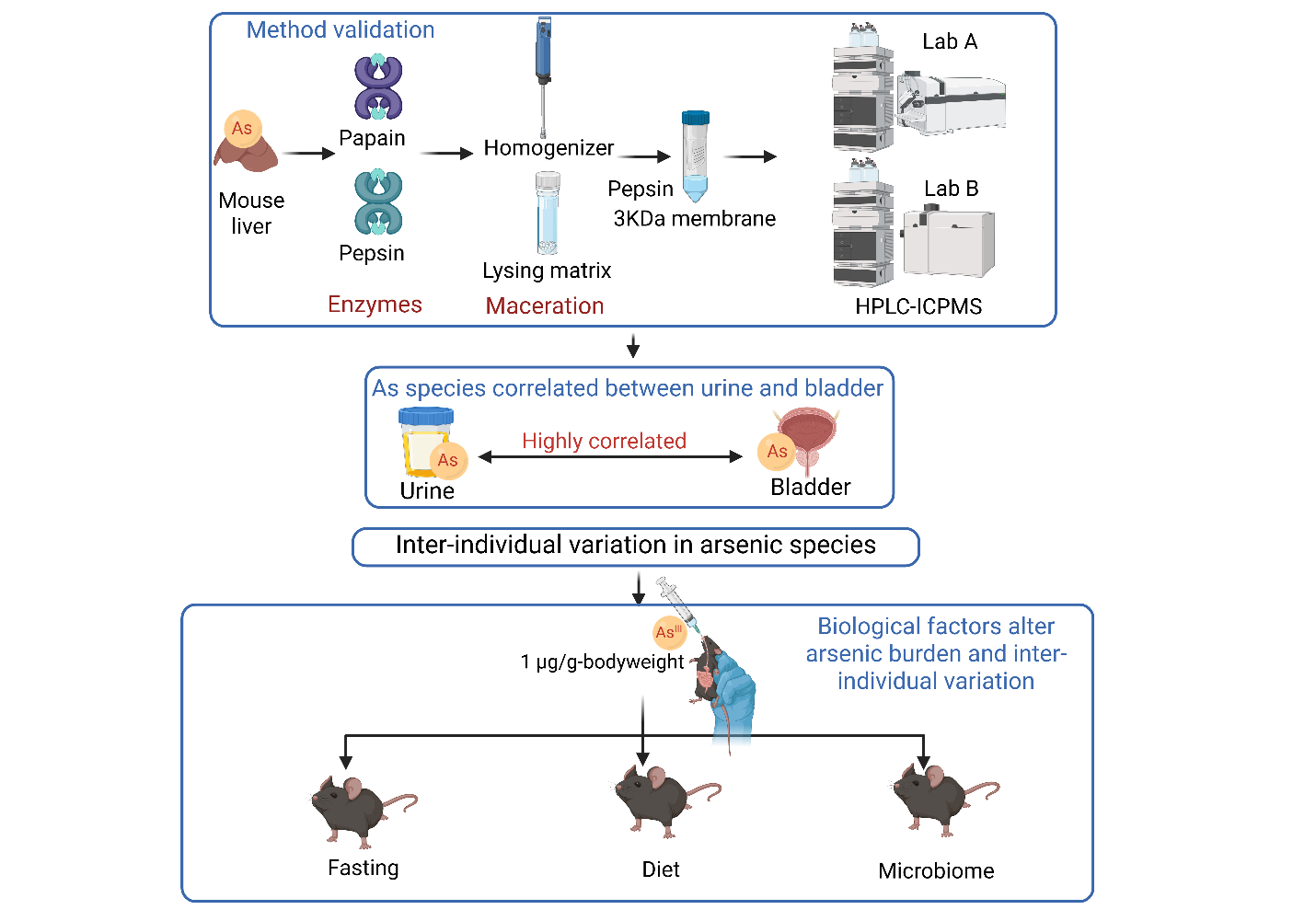


**Supplemental Figure 2.** Background arsenic levels in commercial bead beat tubes. Four commercial bead beat tubes were compared before and after washing with strong acid (multiple unpaired t test with Welch’s correction and FDR correction for multiple comparisons).


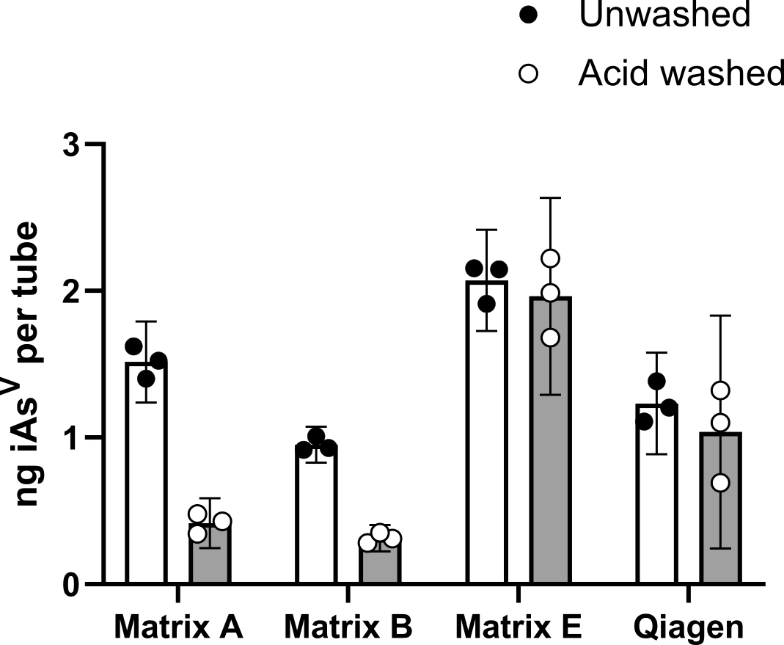


**Supplemental Figure 3.** Arsenic species were compared in liver samples prepared by bead beating or mechanical homogenizer in three different mice following controlled exposure to iAs^III^ (circles represent n=3 technical replicates from the same liver). Graph label is the same for all panels as shown in the top panel.


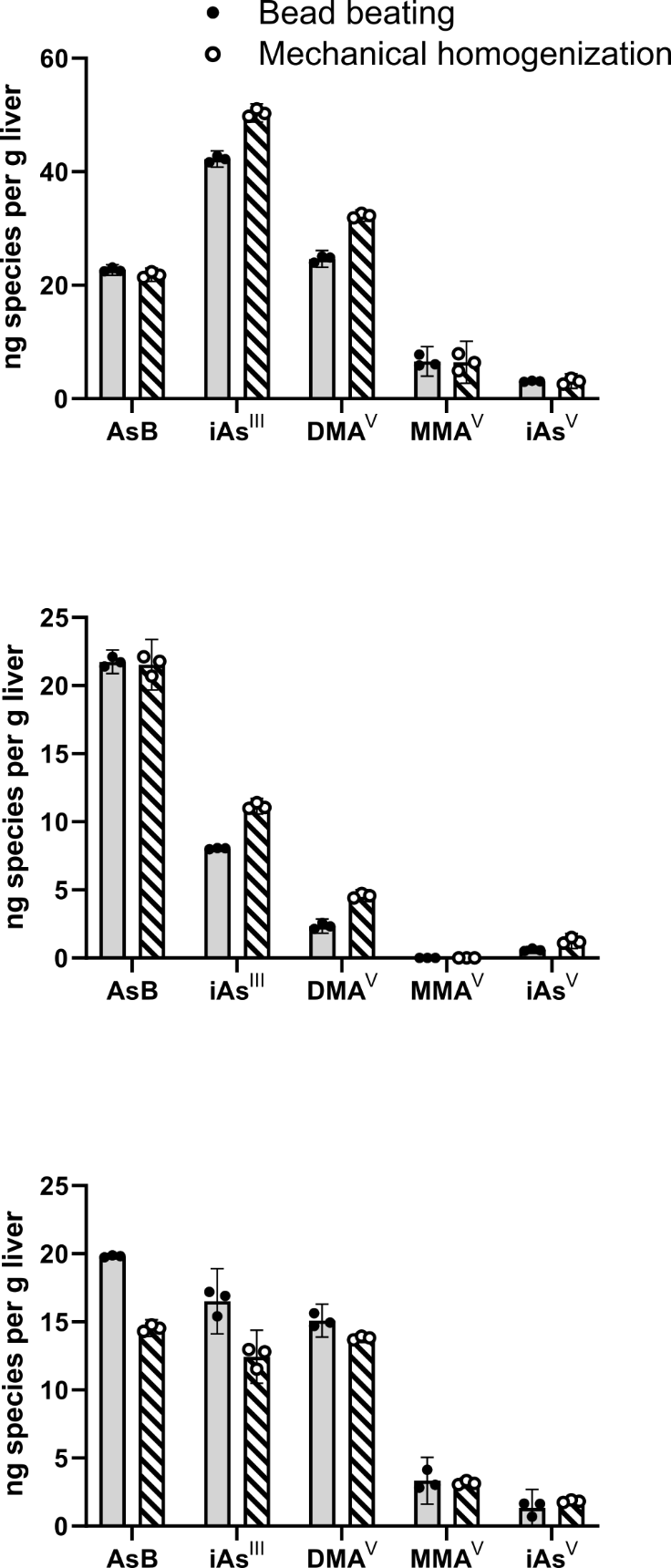


**Supplemental Figure 4.** Relationship between arsenic species levels in urine and bladder tissue samples. Samples containing up to five arsenic species were obtained at necropsy from mice at baseline and every two hours during a defined iAs^III^ exposure (1 µg iAs^III^ per g bodyweight via oral gavage) for eight hours (n=2 mice per time point; 5 time points; n=10 total mice). Raw values were not normally distributed so the relationship was analyzed using Spearman’s rank correlation. Panels A-E correspond to AsB, iAs^III^, DMA^V^, MMA^V^, and iAs^V^, respectively.


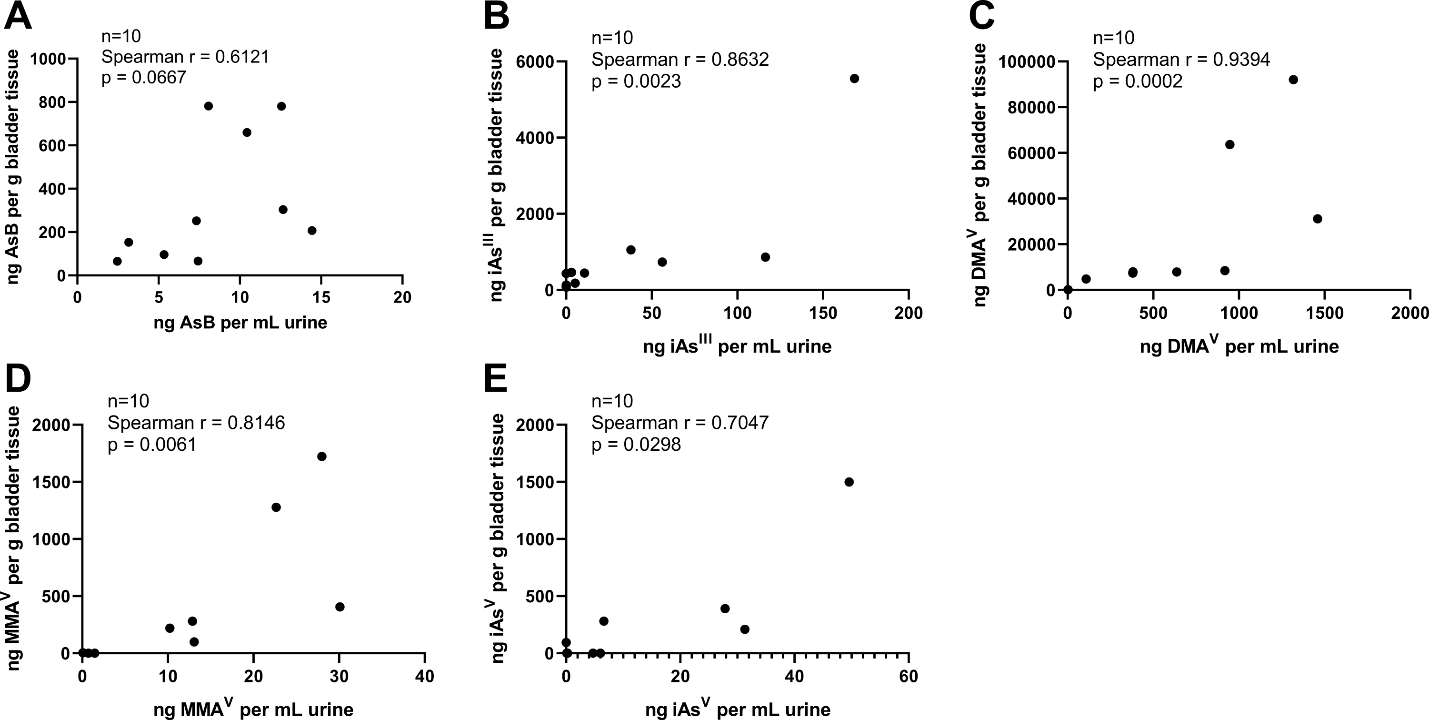


**Supplemental Figure 5.** Side-by-side comparison of representative HPLC-ICPMS profiles from Lab A (PRP-X100 column) and Lab B (CE and C18 columns).


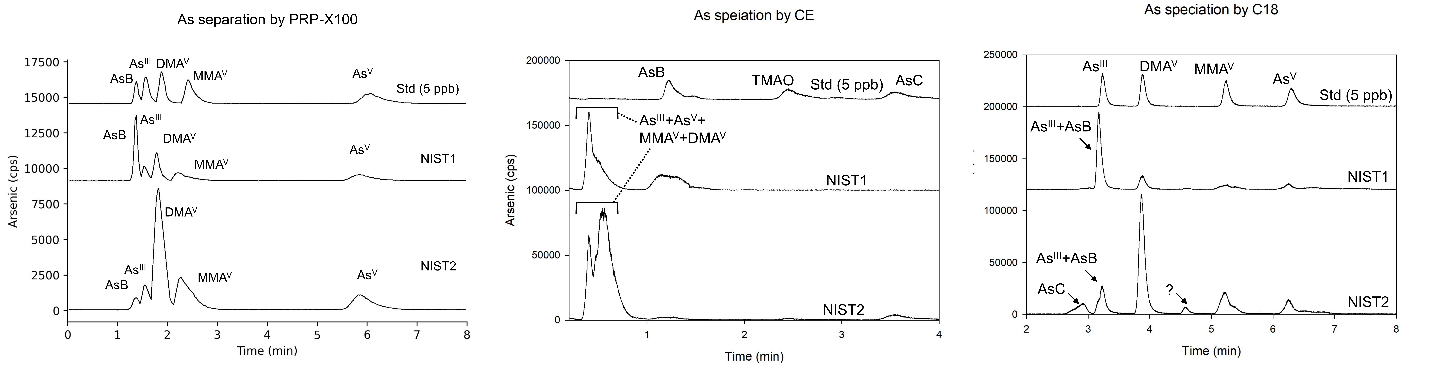


**Supplemental Figure 6.** Arsenic species levels determined by HPLC-ICPMS using two different set-ups and columns (Lab A – anion exchange, PRP-X100, column; Lab B – cation exchange, CE, and C18 reverse-phase columns). Coefficient of variation reported as (standard deviation / mean) * 100%.


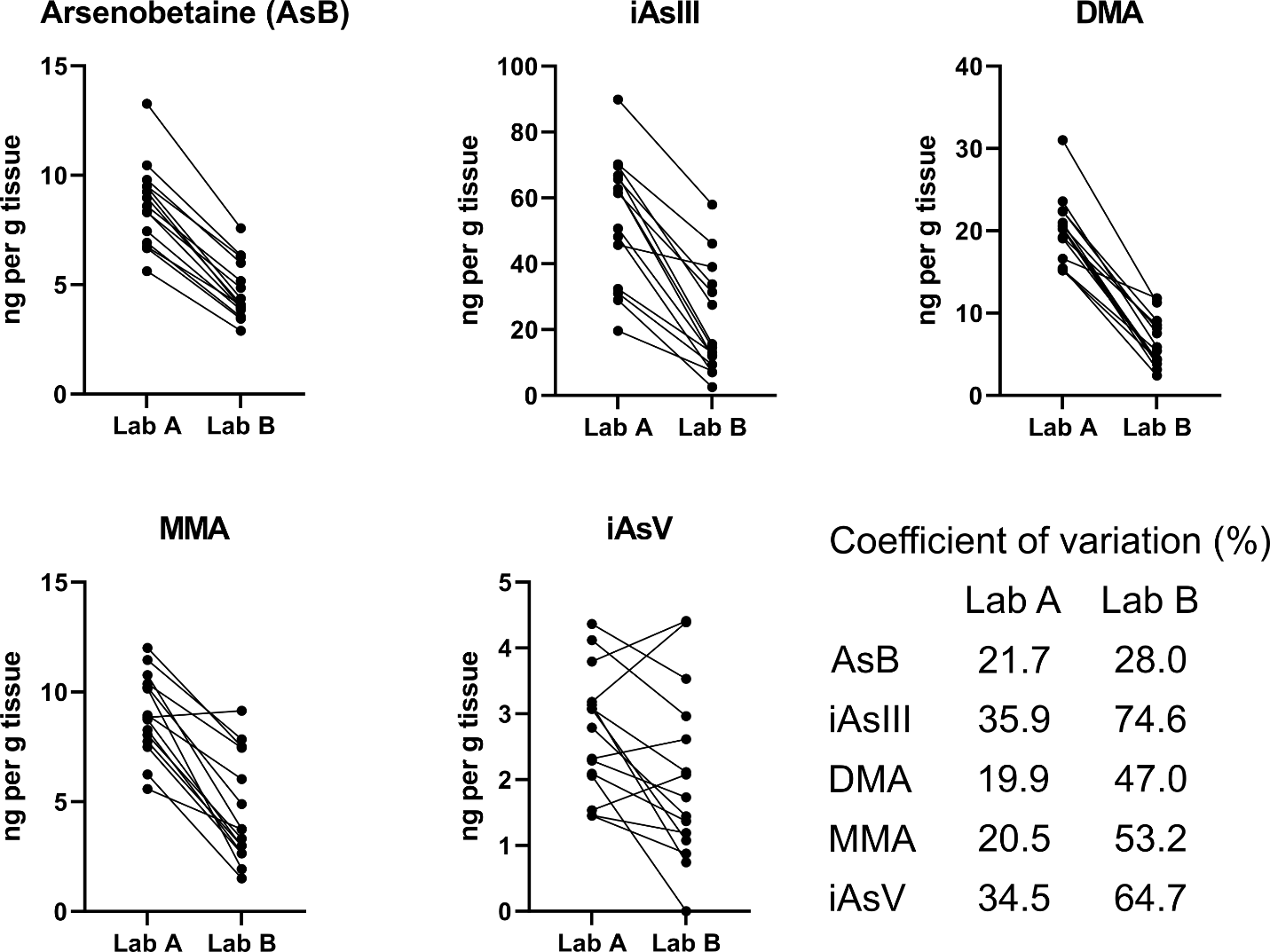


**Further discussion on the potential impact of bead beating lysing matrix on arsenic speciation.**

The apparent loss of iAs^III^ and gain of iAs^V^ in the spike-recovery experiments (Fig. 2B) derives primarily from release of iAs^V^ loosely bound to the beads; simple extraction of beads with ultrapure water resulted in the release of ppb levels of iAs^V^, showing that the manufactured beads carry measurable iAs^V^. Further iAs^V^ release occurs via exchange of the spiked iAs^III^ for bound iAs^V^. ICP-MS analysis of matrix B beads (HCl/HNO_3_ acid extract, see methods) found considerable levels of aluminum (7.7 ± 0.3 μg/tube, mean ± SD, n = 2) and iron (7.8 ± 0.04 μg/tube. Assuming these metals are at least partially in the oxide form, then there would be opportunity for background adsorption of arsenic(Giles et al. 2011; Mercado-Borrayo et al. 2014), particularly iAs^V^, during bead manufacturing. Additionally, appreciable manganese (0.123 ± 0.004 μg/tube) was also found in the ICP-MS analysis. Manganese oxides are well known as strong naturally occurring oxidants(Post 1999; Ying et al. 2012), in particular of arsenic (Fischel et al. 2024), potentially offering an opportunity for oxidation of the spiked iAs^III^.

**Further discussion on arsenic species levels in urine versus bladder tissue.**

Urine capture can be facilitated by the use of metabolic chambers, but with some drawbacks: mice need to be removed from their standard cage environment, urine is collected over a period of time (hours), and typically more than one animal is needed to collect enough volume for analysis. The relationship between arsenic levels in metabolic chamber-collected urine and necropsy-collected bladder tissue samples from arsenic-exposed mice has been evaluated(Kenyon et al. 2008). While similar arsenic species in that study were observed in both urine and bladder samples, the distribution of species differed over the course of the 12-week study, with authors commenting that results are relevant to longer term (“subchronic”) tissue accumulation. This study also used a different detection technology (hydride generation atomic absorption spectrometry, HG-AAS) and did not conduct a correlation analysis to understand whether arsenic species levels correlated at the individual mouse level.

**Further discussion on the impact of different diets during arsenic exposure.**

Carmean *et al*. quantified total arsenic in the liver of mice fed either a purified diet or a high-fat diet after 16 weeks of chronic exposure to iAs^III^ (50 mg/L) in drinking water(Carmean et al. 2020). Interestingly, they observed “dramatic interanimal variations”, which is consistent with our results. No comparisons of arsenic levels between mice eating different diets were reported and it is unclear how dietary fat directly and/or indirectly influences arsenic body burden. We are also unaware of attempts to quantify arsenic levels in murine models that mimic human diets beyond a “high-fat” diet, or especially in studies that experimentally controlled for different human diet components. More importantly, it is unclear whether direct interactions between arsenic and dietary components explain differences in tissue accumulation or whether other interactions (e.g., diet-microbiome-host) play a role.

**Further discussion on the impact of fasting.**

In their evaluation of C57BL/6J mice, Moro and Morgan concluded that two-hour fasting was sufficient to reduce blood glucose variability and reduce insulin levels by 40%, similar to longer (≥ 4-hour) fasting times (i.e., the 3-hour fasting used here is a minimum known to minimize variability of a diet-derived metabolite) (Moro and Magnan 2025). Fasting times >4 hours were associated with time-dependent body-weight loss and altered catabolism in the liver and skeletal muscle (Moro and Magnan 2025), resembling a starvation state. So while our results are relevant to food/water withdrawal that normalize some metabolites, like glucose, it is possible that an increased state of starvation (i.e., fasting >4 hours) would yield different outcomes, potentially due to an altered metabolic state in the liver.

**Further discussion of microbiome impacts.**

Of the three factors evaluated in this study (diet, fasting, and the microbiome), only the microbiome had a significant impact on inter-individual levels of arsenic in tissue. Because the microbiome is more challenging to control compared to diet and other aspects of animal husbandry, it should be expected that results from mice housed in different vivariums will differ due to the presence/absence of different gut microbes. This provides rationale for evaluating the impacts of different human microbiome taxa that either introduce or minimize variability in arsenic levels between individuals. In doing so, risk can be associated with such taxa and tested using epidemiological studies of exposed human populations.
